# Copper Catalysts Inherit and Retain Precatalyst Morphology in Extended CO Electroreduction to *n*‐Propanol

**DOI:** 10.1002/adma.202508900

**Published:** 2025-08-23

**Authors:** Ji‐Yoon Song, Jianan Erick Huang, Hyeong Woo Ban, Qiu‐Cheng Chen, Yali Ji, Shuang Yang, Yong Wang, Yunsung Yoo, Hyun Seung Jung, Jiachen Li, Heejong Shin, Ke Xie, Edward H. Sargent

**Affiliations:** ^1^ Department of Chemistry Northwestern University Evanston IL 60208 USA; ^2^ Department of Electrical and Computer Engineering Northwestern University Evanston IL 60208 USA

**Keywords:** C_1_‐C_2_ coupling, CO_2_ and CO reduction, electrosynthesis, grain boundaries, n‐propanol, precatalyst engineering, rapid heating and cooling

## Abstract

Copper catalyst morphology, faceting, and oxidation state are each known to impact selectivity in the electroreduction of CO. Copper oxide precatalysts are synthesized using flash Joule heating and rapid cooling, and it is observed that temperature ramp rates can be used to control morphology, enabling us to implement ≈10 nm‐sized intragrain features within ≈35 nm grains. It is found that the structural features of the precatalysts are substantially transferred to Cu catalysts that are formed when they are employed in CO electroreduction in a membrane electrode assembly electrolyzer. The catalysts achieve ≈35% faradaic efficiency to *n*‐propanol, among the highest selectivities to C_3_ from monometallic Cu. Both selectivity and morphology are retained following 330 h of operation at 100 mA cm^−2^. CO dilution studies reveal that catalysts with similar faceting, but smaller grain sizes, exhibit *n*‐propanol selectivity that increases with CO concentration, suggesting that grain interfaces contribute to CO coverage and C_1_–C_2_ coupling. Complementary *operando* Raman spectroscopy shows that reducing grain size enables higher CO coverage, suggesting that structural features enhancing linear CO adsorption are correlated with improved selectivity to C_3_.

## Graphical abstract

Flash Joule heating enables the formation of quenched Cu oxide precatalysts with intragrain‐in‐grain architectures. These hierarchical structures are inherited after electroreduction, suppressing reconstruction even at high current densities in an MEA cell. The high intragrain boundary density enhances CO adsorption and C_1_–C_2_ coupling, leading to selective n‐propanol formation ≈35%. This approach achieves both structural durability and improved C_3_ selectivity.

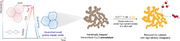

## 1 Introduction

Electrochemical CO and CO_2_ reduction reactions (CORR and CO_2_RR) offer routes to multicarbon chemicals.^[^ ^1^ ^]^ Among catalyst systems, copper‐based materials conduct to C–C coupling, consistent with their moderate binding strength to *CO intermediates.^[^ ^2^ ^]^ Cu oxides such as Cu_2_O and CuO are often employed as precursors since, under applied reducing potentials, they produce active sites prone to C_2+_ product formation.^[^ ^3^ ^]^ These oxide‐derived Cu catalysts undergo reconstruction, including in gas‐fed membrane electrode assembly (MEA, zero‐gap configuration) electrolyzers, which operate at high current densities. Some precatalysts have been seen to collapse into nanodebris or to coarsen into larger grains.^[^ ^4^ ^]^ This extreme disruption to morphology presumably severs any strong link between the nature and density of grains and grain boundaries in the precatalyst and the morphology of the *operando* copper catalyst.

Numerous reports find that precatalysts can influence the structure/morphology, and correspondingly the selectivity/performance, of the operating catalyst^[^ ^4^, ^5^ ^]^—a phenomenon we refer to as template transfer. In precatalyst syntheses, such as hydrothermal and electrodeposition, that involve the nucleation growth of copper oxide particles, grains initially nucleate as small crystallites during the early stages of heat treatment, and tend to grow into larger grains over time.^[^ ^6^ ^]^ This grain growth is driven by increased atom mobility and the reduction of interfacial energy, and grains merge into more thermodynamically favorable configurations, often exhibiting aligned crystallographic facets.^[^ ^7^ ^]^ This coarsening reduces the density of grain boundaries—structural features known to promote CO adsorption and C–C coupling pertinent to making C_3_ products.

Preserving the link between precatalyst and final catalyst morphology/structure has been enhanced by synthesis approaches that increase precursor–electrode contact, prevent grain detachment, and minimize morphological disruption during electroreduction.^[^ ^8^ ^]^

**Figure 1:**
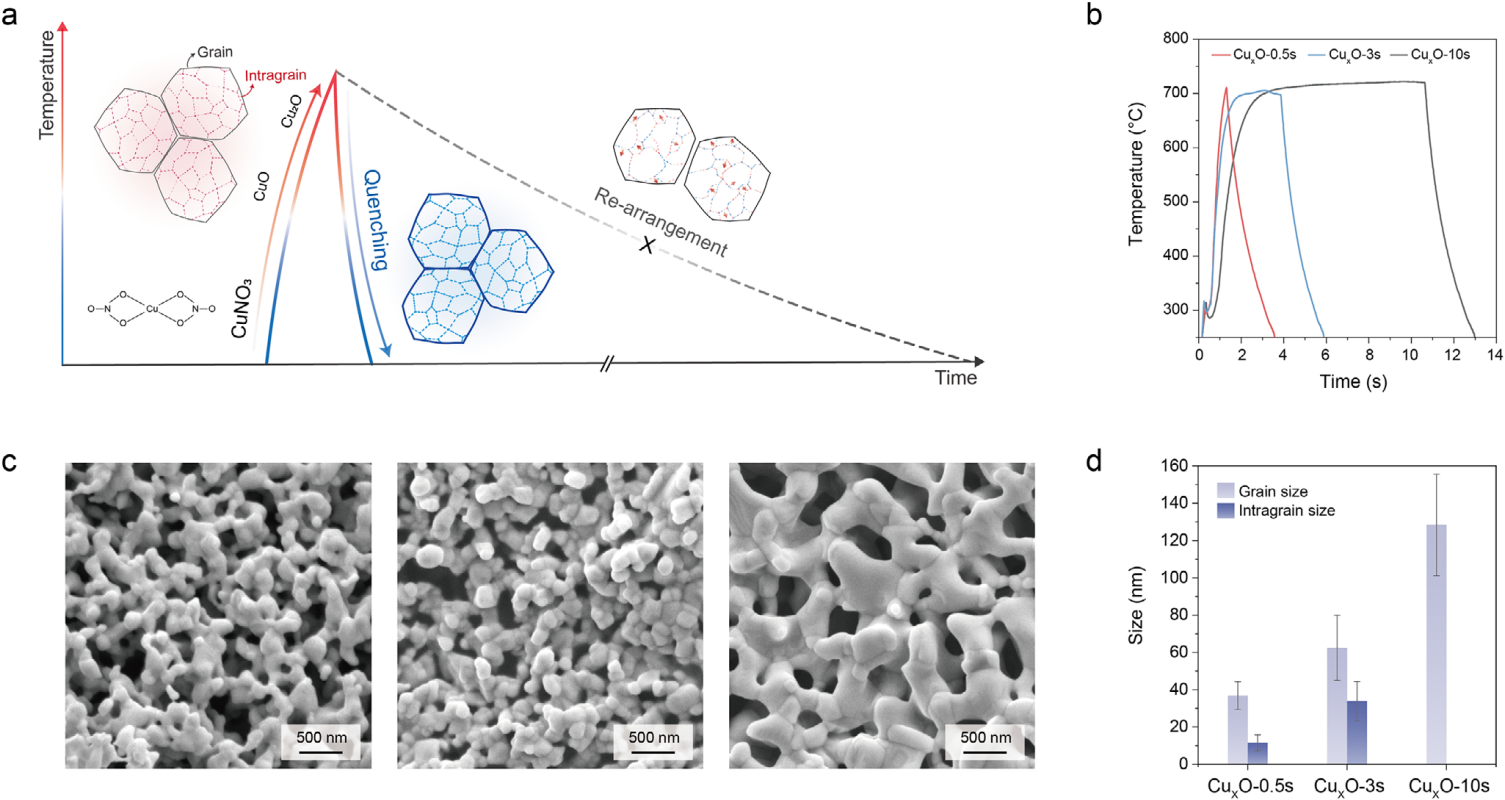
Cu oxide (Cu_x_O) precatalyst grain structures formed under Joule heating. a) Schematic showing flash heating and fast quenching. b) Time‐temperature profiles for Joule heating‐assisted synthesis. c) SEM images of Cu_x_O precatalysts synthesized depending on heating time (0.5, 3, and 10 s). d) Grain and intragrain sizes for Cu_x_O‐*xs*.

We hypothesized that the precatalyst synthesis could be further refined with the goal of increasing the fidelity of template transfer, and of better defining the precatalyst whose morphology is to be inherited in the operando catalyst. Our approach was to decrease grain size and increase grain boundary density by forming copper oxide precatalysts via flash Joule heating. We find herein that accelerating this heating and cooling process enables sintering and oxidation of the Cu precursor, CuNO_3_, in such a way that a more interconnected catalyst network is provided, and robust interparticle contact is achieved (**Figure** **1**).

## 2 Results and Discussion

To create a higher density of grain boundaries with a small grain size, we employed a rapid heating/cooling‐based synthesis to prepare Cu oxide (Cu_x_O) precatalysts. When the catalyst is cooled swiftly, a non‐equilibrium/metastable structure can become “frozen” into the final material–the precatalyst in our study. This approach kinetically traps a hierarchical structure—featuring nanoscale intragrain domains embedded within confined grains by rapid quenching. We apply flash heating followed by rapid cooling (≈205 K s^−1^) to limit atomic rearrangement during grain evolution. Our ultimate purpose is to yield oxide structures with suppressed facet alignment and enhanced boundary heterogeneity.^[^ ^9^ ^]^

**Figure 2:**
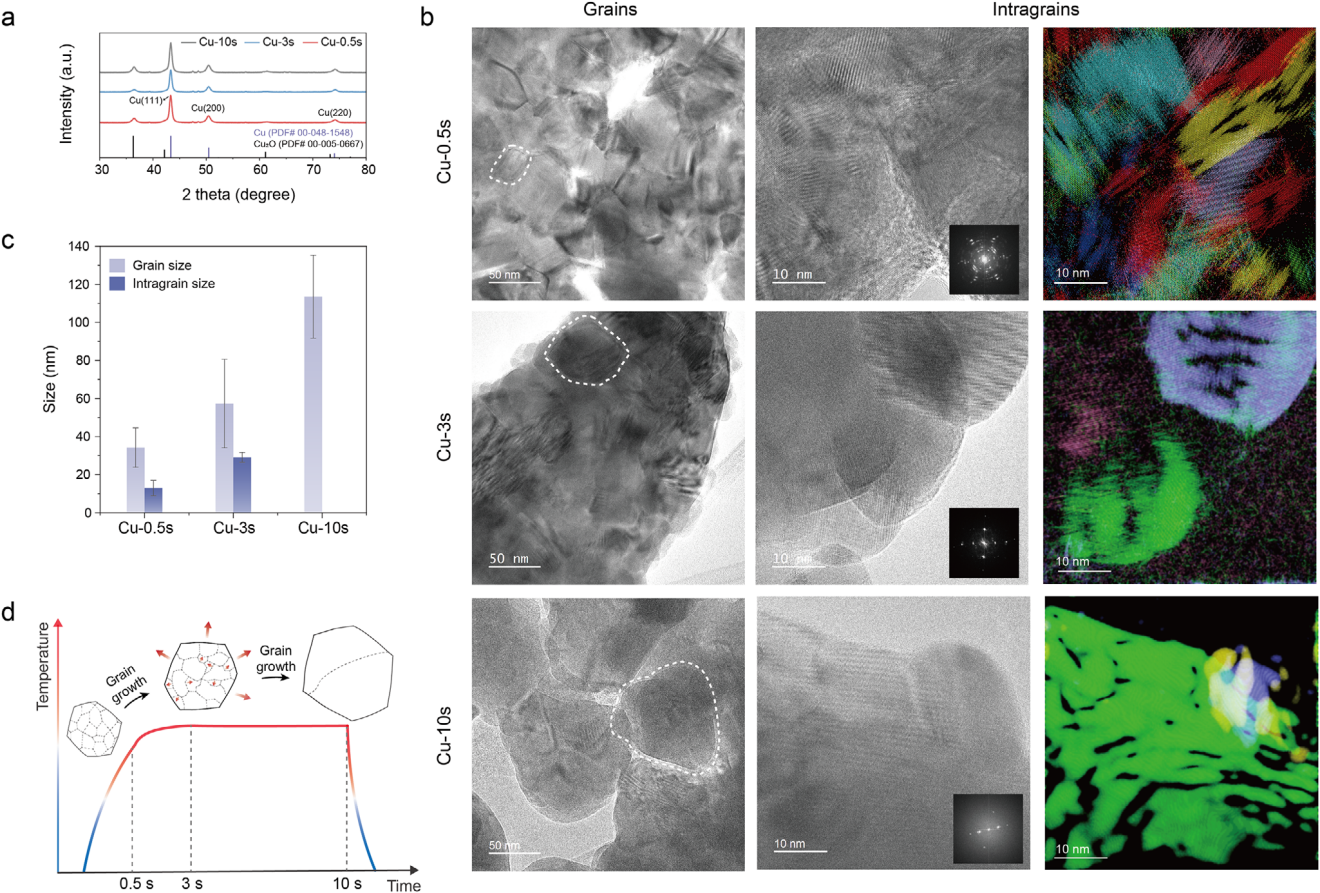
Characterization of metallic copper catalysts, Cu‐0.5s, 3s, and 10s, following electroreduction. a) XRD patterns, b) TEM images and corresponding Cu grains (inset: FFT patterns), c) Grain and intragrain size for Cu‐*x*s, and d) illustration for grain growth depending on synthesis time.

To evaluate the effect of heating time on precursor structure, we synthesized Cu_x_O samples using heating times of 0.5, 3, and 10 s at 700 °C (Figure 1b). To construct an interconnected catalyst framework that minimizes reconstruction during electroreduction, we optimized the heat‐treatment temperature. SEM results (Figure S1, Supporting Information) revealed that such interconnected features were not formed below 700°C; whereas sintered, continuous structures emerged at 700°C. SEM images in Figure 1c reveal that prolonged heating leads to grain coarsening: Cu_x_O‐0.5s showed sub‐100 nm grains with irregular contours, while Cu_x_O‐10s displayed larger and more consolidated grains (200–500 nm). We conducted high‐resolution transmission electron spectroscopy (HR‐TEM) to evaluate grain growth behavior as a function of heating time. TEM analysis of the Cu oxide precatalysts revealed significant structural grain variation that depends on heating time (Figure 1d; Figures S2–S6, Supporting Information). The Cu_x_O‐0.5s sample consisted of grains with an average size of 37 ± 7 nm and intragrain domains of 12 ± 4 nm. Cu_x_O‐3s showed moderate coarsening with grain sizes of 62 ± 17 nm and intragrains of 34 ± 11 nm. The Cu_x_O‐10s sample exhibited the largest grains at 130 ± 30 nm, with the disappearance of intragrain domains, attributed to their growth under prolonged heating time (Further details are discussed in **Figure** **2**). XRD patterns indicate that all samples exhibit mixed‐phase compositions of Cu_2_O and CuO, although the relative phase ratios vary (Figure S7, Supporting Information).

These oxide precatalysts are electrochemically reduced to metallic Cu under CO electroreduction conditions. Notably, the CuO made with a faster cooling generates smaller and more evenly distributed grain boundaries in the final copper catalyst: Cu‐0.5s retained fine grains and distinct intragrain, while Cu‐10s underwent grain growth and exhibited aligned crystalline facets (Figure 2).

This observation motivated a deeper investigation into how the degree of grain coarsening prior to reduction correlates with catalytic performance in membrane electrode assembly (MEA) systems. We conducted post‐CO‐electrolysis structural characterization, aiming to identify structure–function relationships correlated with the precatalyst.

To probe the structural characteristics of the active electrocatalyst state during CORR, we conducted electroreduction at −100 mA cm^−2^ for 15 min (Figure S8, Supporting Information), and then carried out ex situ characterization. After electroreduction, most of the copper oxides were reduced to metallic Cu, with only minor residual CuO(111) peaks at 36.5° (Figure 2a; Figure S9, Supporting Information). The relative ratios of Cu(111), Cu(200), and Cu(220) facets remained similar across samples, independent of the precursor synthesis time (Figure S10, Supporting Information). As a result, in our later analysis correlating materials synthesis conditions with catalytic performance, we argue that influences other than faceting may be important determinants of catalytic performance. TEM analysis of the post‐operando Cu catalysts shows significant structural variation that depends on the heating time employed in precatalyst synthesis (Figure 2b). The Cu‐0.5s sample exhibited fine grains 34 ± 10 nm with multiple lattice orientations coexisting within individual grains—features which we term intragrains (Figures S11–S16, Supporting Information).

We offer that–in precatalyst synthesis–the rapid heating–cooling process leads to limited crystallographic alignment, and that this produces structurally heterogeneous grains exhibiting a high internal boundary density. It is striking that, in the post operando catalyst, these complex grain architectures are retained. We propose that the structural motifs initially embedded in the precatalyst may be inherited by the metallic Cu phase. When, in precatalyst synthesis, we employed much more extended heating (CuO‐10s, which becomes Cu‐10s), the post operando catalyst manifests grain coarsening and a progressive reduction in intragrain heterogeneity, consistent with thermally driven crystallographic ordering within the precatalyst. FFT analysis highlights this trend: Cu‐0.5s showed multiple diffraction rings within single grains, whereas Cu‐10s displayed sharp, monocrystalline patterns indicative of orientational uniformity (Figure 2b, inset).

As summarized in Figure 2c, the Cu‐0.5s sample exhibited an average grain size of 34 ± 10 nm and intragrain domains of 13 ± 4 nm. Cu‐3s showed moderate coarsening with grain sizes of 57 ± 23 nm and intragrains of 29 ± 3 nm. Cu‐10s exhibited fully coalesced grains (110 ± 20 nm) with no observable intragrains, reflecting advanced crystallographic reorganization through grain merger and elimination of energetically less‐stable domains.^[^ ^6^, ^7^ ^]^ The ability to influence, and to verify operando, the Cu facet ratios and the grain/intragrain structures, provides a means to correlate grain boundary configurations with catalytic performance.

**Figure 3:**
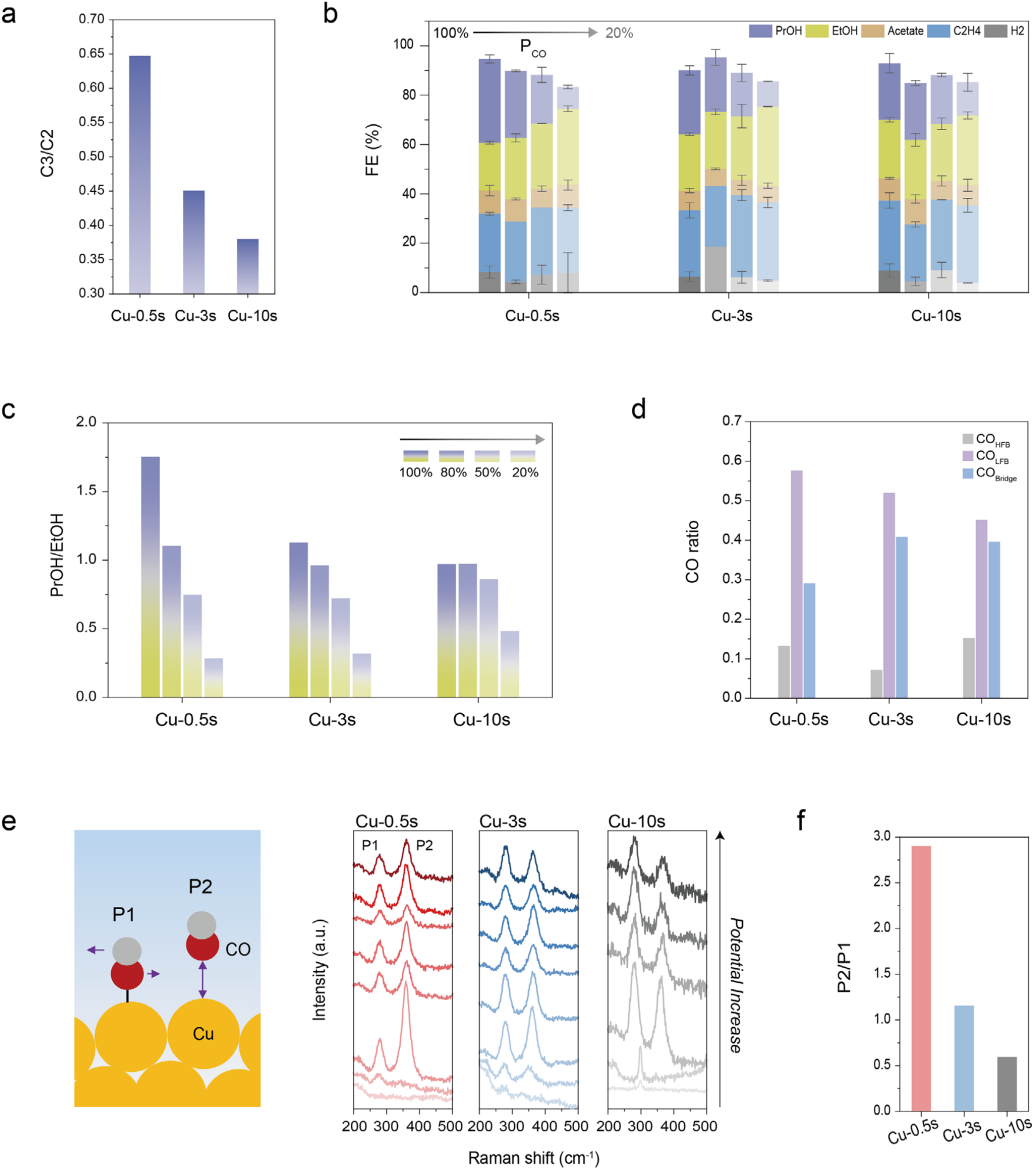
CORR performance of Cu‐0.5s, 3s, and 10s electrocatalysts in MEA electrolyser and operando Raman studies. a) C_3_/C_2_ ratio depending on grain size b) FEs for Cu‐*x*s under different CO partial concentration from 100% to 20%. c,d) PrOH/EtOH under different CO partial concentration. d) C–O stretching band deconvolution in the range of 1900–2100 cm^−1^ in Raman spectra (CO_HFB_: high‐frequency band linear CO and CO_LFB_: low‐frequency band linear CO) e) Raman spectra for adsorbed CO during CORR from −1.0 to −1.9 V versus RHE and corresponding f) P2/P1 ratio.

To evaluate catalytic behavior, we conducted CO electroreduction in a membrane electrode assembly (MEA) system using alkaline anolyte and operating at a constant current density of 100 mA cm^−2^. As summarized in **Figure** **3**a, the C_3_/C_2_ product ratio increased with decreasing grain size. Cu–0.5s exhibited the highest C_3_/C_2_ ratio (≈0.65), followed by Cu‐3s (≈0.45) and Cu‐10s (≈0.38), consistent with the trend in grain size.^[^ ^5^, ^10^ ^]^ Previous theoretical and experimental studies have found that grain boundaries serve as favorable sites for *CO adsorption and C_1_–C_2_ coupling, facilitating the formation of C_3_ products.^[^ ^10^, ^11^ ^]^ Higher reactant coverage has been observed to correlate positively with the C_1_–C_2_ coupling pathway necessary to produce n‐propanol.^[^ ^12^ ^]^ We therefore took the perspective that C_3_ product formation is likely favored by having sufficient *CO surface coverage, as well as by the presence of high‐density grain interfaces that co‐localize C_1_ and C_2_ intermediates. The first provides the needed C_1_ reactants, and the second the spatial proximity necessary for coupling to occur.

To investigate the effect of CO surface coverage on product distribution, we modulated CO concentration by diluting with inert Ar gas (Figure 3b). For Cu‐0.5s, which achieved an n‐propanol FE of ≈35%—one of the highest reported among monometallic Cu catalysts (Figure S17, Supporting Information)—the selectivity exhibited a marked shift with decreasing CO concentration, with a diminishing C_3_/C_2_ ratio when we decreased CO concentration in the gas mixture. In contrast, Cu‐3s shows similar trends but a less significant shift in the C_3_/C_2_ ratio, and Cu‐10s shows only minimal changes in selectivity across CO partial pressures (Figure 3c; Figure S18, Supporting Information). This agrees with the picture in which CO coverage enables C_1_–C_2_ coupling on a Cu surface having smaller grains and higher grain boundary densities (Figure S19, Supporting Information).^[^ ^13^ ^]^

We also probed CO adsorption behavior and its correlation with structure using *operando* Raman spectroscopy (Figure 3d–f; Figures S20–S22, Supporting Information). Vibrational features at ≈280 and ≈365 cm^−1^ correspond to the hindered rotation of adsorbed CO (P1) and the Cu–CO stretching mode (P2), respectively. The area ratio of P2 to P1, which serves as an indicator of surface CO coverage,^[^ ^14^ ^]^ varies significantly with potential and among the three catalysts studied (Figure 3e). Cu‐0.5s samples exhibit the highest P2/P1 ratio beginning from the onset of CO reduction, indicating the highest CO coverage (Figure. 3f). The grain size distribution—while maintaining a similar Cu facet ratio—influences CO adsorption characteristics on the catalyst surface. To analyze the CO adsorption sites on the Cu, we deconvolute the C─O stretching band (1900–2100 cm^−1^) to three distinct components: the low‐frequency band (CO_LFB_), high‐frequency band (CO_HFB_), and CO adsorbed at bridge sites (CO_bridge_).^[^ ^15^ ^]^ A higher fraction of CO_LFB_ is associated with CO bound to more coordinated Cu sites, which has been found to favor C–C coupling, including *CO dimerization (C–C) and C_1_–C_2_ coupling.^[^ ^16^ ^]^ We analyze the CO adsorption sites on three catalysts at the potential where CO coverage reached maximum according to the P2 to P1 ratio (1.4–1.5 V vs. RHE): Cu‐0.5s showed the highest CO_LFB_ fraction (58%), followed by Cu‐3s (52%) and Cu‐10s (45%) (Figure 3d).

Thus, Cu‐0.5s, with small grain size and high density of grain boundaries, facilitates greater CO surface coverage, with CO preferentially adsorbing in linear low‐frequency binding configurations, enabling a more highly‐coordinated environment that fosters the desired Cu sites for C─C and C_2_‐C_1_ coupling in the context of high CO coverage. This agrees with the CO dilution studies, which found that the higher density of grain boundaries led to performance that is more sensitive to the increase in CO coverage in influencing CORR selectivity.^[^ ^12^ ^]^ Overall, this picture–in which high grain boundary density increases local *CO coverage and enables spatial co‐localization of C_1_ and C_2_ intermediates–appears consistent across this suite of studies.^[^ ^17^ ^]^

**Figure 4:**
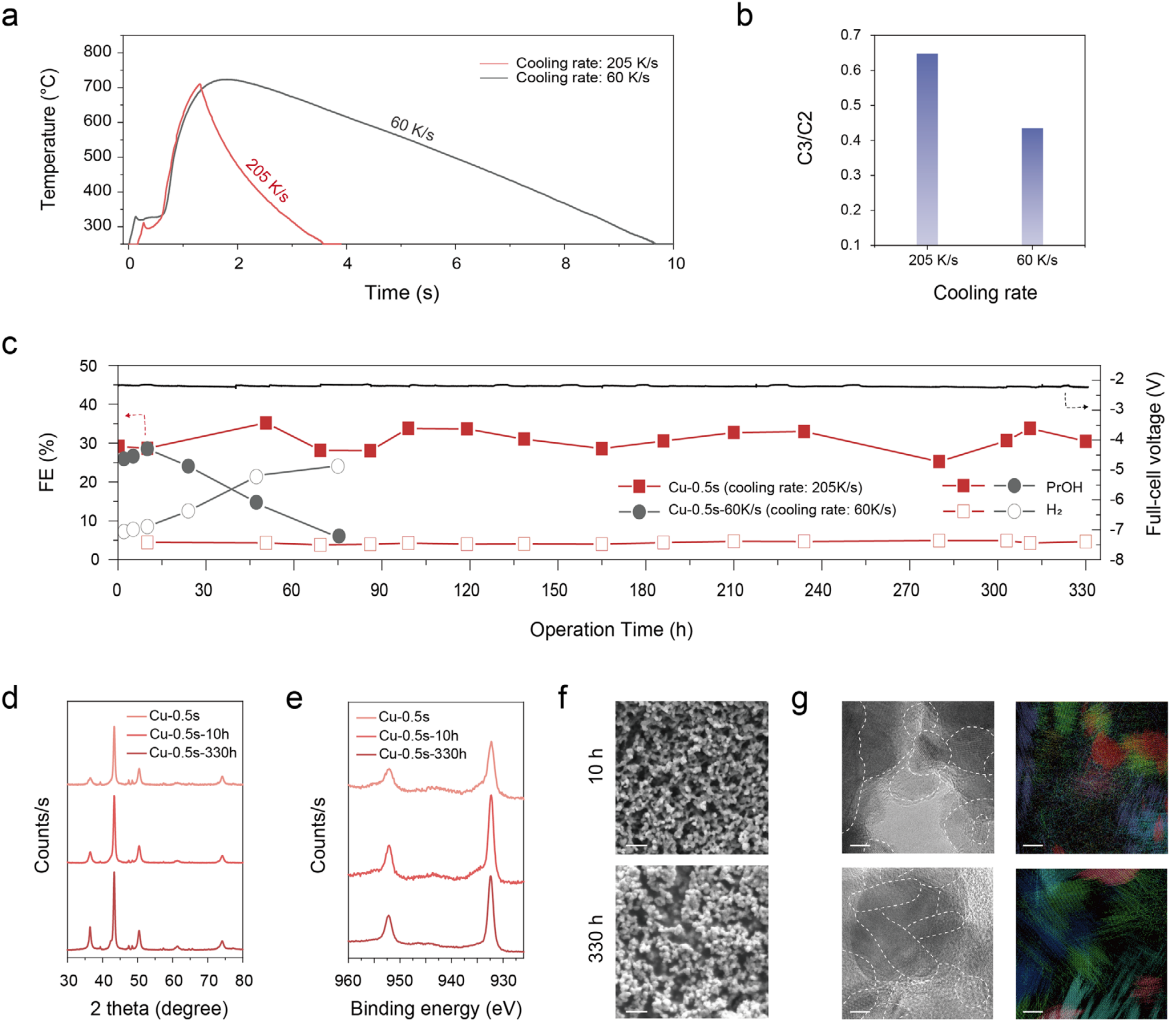
Effect of cooling rate on catalyst stability and post‐electrolysis structure of Cu‐0.5s. a) Temperature profiles, b) C_3_/C_2_ product ratio, c) >330 h of stability under −100 mA cm^−2^ in MEA electrolyser. d) XRD patterns, e) Cu *2p* XPS high‐resolution spectra, f) SEM images (scale bars: 500 nm), and g) TEM images after stability test of Cu‐0.5s (scale bars: 5 nm).

To investigate the impact of thermal quenching on long‐term catalyst stability and structure following CO electroreduction, we then compared the performance and post‐operation morphology of Cu‐0.5s catalyst subjected to different cooling rates (**Figure** **4**). We slowed the cooling rate to 60 K s^−1^ to test the role of quenching rate. The catalyst corresponding to the Cu‐0.5s precatalyst made using the slower cooling rate was formed by progressive decrease in the current following stepwise Joule heating (Figure 4a). As illustrated in Figure 1a, during the slow cooling process, there is a greater extent of grain realignment, resulting in a relatively irregular interconnected morphology (Figure S23a, Supporting Information) and a larger average grain size of ≈50 nm (Figure S23b, Supporting Information). This structure exhibited a lower C_3_/C_2_ product ratio compared to the Cu‐0.5s catalyst, yielding results comparable to those of Cu‐3s (Figure 4b; Figure S24a, Supporting Information).

In addition to the structural changes induced by the slow cooling rate, the electrocatalytic stability under CORR operation was also evaluated. When we operated Cu‐0.5s and Cu‐0.5s‐60K s^−1^ at 100 mA cm^−2^ in MEA (Figure 4c), the faradaic efficiency (FE) of Cu‐0.5s to *n*‐propanol remained stable at ≈30% over a 330 h continuous testing period. This reaction, which was coupled with OER on the anode, began at full cell voltage 2.15 V and finished at 2.22 V, an increase rate of ≈1 mV h^−1^. In contrast, in the absence of the grain quenching effect induced by ultrafast cooling, the FE of propanol decreased to 15% within 47 h, and the full cell voltage increased at a rate of ≈2 mV h^−1^ (Figure S24b, Supporting Information). Additionally, the slowly cooled Cu‐0.5s (Cu‐0.5s‐60K s^−1^) exhibited changes in the selectivity of gas products during the initial 10 h of CORR operation (Figure S25, Supporting Information). We witnessed similar “mapping” of precatalyst morphology onto post‐10‐h‐in‐MEA morphology in the case of Cu‐3s and Cu‐10s as well (Figures S26–S29, Supporting Information). These observations highlight the role of quenching in preserving catalyst stability and morphology under prolonged CO electroreduction operation.

We studied the catalyst after 330 h runtime (Figure 4d–g) and found from XRD that metallic Cu (111), (200), and (220) remained consistent with the patterns observed after 1 h (Figure 4d). Cu *2p* XPS spectra were also similar for 1 versus 330 h, with 932.3 and 952.0 eV peaks indicative of metallic Cu species (Figure 4e; Figure S30, Supporting Information). From SEM, the interconnected Cu morphology with ≈100 nm characteristic features remained largely intact (Figure 4f). From TEM images, the fine‐grain structure and intragrain domains remained similar to those observed before operation; though we did detect a slight increase in facet orientation was observed, suggesting partial reorganization of grain surfaces under extended bias (Figure 4g; Figure S31, Supporting Information). Notably, lattice fringes associated with Cu_x_O species appeared on the surface, though this could arise from reoxidation in an air ambient post‐operation. Significant residues of structural features initially encoded into the precatalyst were retained through extended MEA operation.

Collectively, the observed structural persistence, including the retention of small grains and intragrain domains, correlates with the extended selectivity toward *n*‐propanol under MEA conditions. While partial reorganization and surface oxidation were observed, the morphological and chemical integrity of the Cu‐0.5s electrode was sufficient to sustain catalytic selectivity over 330 h.

In this work, we investigated the correlation between the C_3_/C_2_ ratio and grain size, and found a strong relationship. We point out that other factors, such as the oxidation state of Cu and the presence of defects, may also vary during synthesis and contribute to changes in the C_3_/C_2_ ratio. Additional analysis and discussion are provided in Figure S19 (Supporting Information).

## 3 Conclusion

In this study, we found that flash Joule heating followed by rapid quenching enables the synthesis of copper oxide precatalysts that exhibit hierarchical architectures composed of nanoscale intragrain domains embedded within confined grains. These nanostructured features are retained after electroreduction in membrane electrode assembly (MEA) systems and enable high selectivity toward n‐propanol, as well as enabling extended operation.

Catalysts derived from precursors with minimized grain growth (Cu‐0.5s) exhibited a high density of grain boundaries, and this contributed to enhanced CO surface coverage and facilitated C_1_–C_2_ coupling. *Operando* Raman spectroscopy and CO dilution experiments suggest that these grain interfaces promote selective C_3_ product formation in a CO‐rich environment. The catalysts retain their structural and functional characteristics over 330 h of continuous operation at 100 mA cm^−2^, achieving one of the highest reported faradaic efficiencies (≈35%) toward *n*‐propanol among monometallic Cu catalysts.

These findings contribute to work on structure–performance correlation, wherein inherited grain and intragrain architectures suppress catalyst reconstruction and improve the spatial proximity and coverage of key intermediates.

## 4 Experimental Section

### Materials

Copper (II) nitrate trihydrate (CuNO_3_·3H_2_O, ≥99.9%, Sigma–Aldrich), methanol, potassium hydroxide (KOH, 99.98%, Thermo Scientific Chemicals), sodium hydroxide (NaOH, ≥98.0%, Sigma–Aldrich), dimethyl sulfoxide (DMSO, ≥99.9% anhydrous, Sigma–Aldrich) and deuterium oxide (D_2_O, 99.9 at% D, Sigma–Aldrich) were used without further purification. MilliQ water (18.2 MΩ·cm) was used during all of the experimental procedures, including membrane cleaning and electrolyte preparation.

### Synthesis of Cu_x_O‐*x*s

A total of 450 µL of a CuNO_3_/methanol solution (6.04 g in 10 mL) was spray‐coated onto a 1.4 cm × 1.4 cm carbon paper (Freudenberg H23, Fuel Cell Store) to load the Cu precursor and dried for 1 h to evaporate residual methanol. The Cu precursor‐loaded carbon paper was connected at both ends using silver paste, and a current was applied by a power supply to induce Joule heating raising the temperature to 700 °C in the Ar‐filled glove box as previous works.^[^ ^18^ ^]^ Joule heating temperature was measured using an infrared thermometer (Optis). The grain size was controlled by varying the Joule heating duration to 0.5, 3, and 10 s, called Cu_x_O‐0.5s, Cu_x_O‐3s, and Cu_x_O‐10s, respectively. And for gas diffusion on the catalyst surface, 70 µL of 5 wt.% Nafion dispersion (D520, Ion power) was drop‐casted onto the Cu_x_O‐*x*s side and dried for a day.

### Synthesis of Cu‐*x*s

To characterize the catalysts during the electroreduction reaction, the samples were electrochemically reduced in an MEA electrolyzer at −100 mA cm^−2^ for 15 min under CO feeding conditions. The three resulting samples from Cu_x_O‐*x*s were named as Cu‐0.5s, Cu‐3s, and Cu‐10s, respectively.

### Electrochemical Measurement

The CORR experiments were carried out using an MEA electrolyser. The MEA electrolyzer was assembled in a zero‐gap configuration, where the cathode, membrane, and anode are in direct contact. For the cathode, Cu‐xs was used as the electrocatalyst for the CORR, while a commercial Ni foam (Fuel Cell Store, 11070049) was employed as the anode for the oxygen evolution reaction. A cation exchange membrane (Nafion 211, Ion Power) was placed between the electrodes to facilitate ion transport. Except for the electrode area, the silicon gasket covered the surface of the MEA to protect the electrode and for electrical insulation. The reaction area was also regulated to 1 cm^2^ by the pore area in the gasket. The serpentine gas‐feeding and liquid‐flow channels were embedded as grooves in the regions where the electrolyzer interfaces with the electrodes. On the cathode side, CO gas was continuously fed at a flow rate of 25 sccm. On the anode side, 1 m NaOH was circulated at a flow rate of 80 mL min^−1^ using a peristaltic pump. A galvanostatic method was used to apply the electrolysis, and the current density was set to −100 mA cm^−2^ using a potentiostat (BioLogic). For the long‐term stability test, the anolyte was replaced every 10–20 h to maintain a consistent cation concentration, as cations are continuously lost to the anode through the CEM.

### Product Analysis

Gas products were sampled in 1 mL increments using gas‐tight syringes (Hamilton chromatography syringe) and subsequently analyzed using an offline gas chromatograph (Clarus 680 GC, Perkin Elmer). The GC system was equipped with two detectors: a flame ionization detector (FID) for quantifying C_2_H_4_ gas and a thermal conductivity detector (TCD) for detecting H_2_ gas. The peaks from gases were calibrated using a standard gas. The resulting spectra from each injection were used to determine the Faradaic efficiencies (FEs) of the gas‐phase products generated during CORR.

Liquid‐phase products were sampled by purging the cathode outlet line into deionized water and the anolyte after operation, respectively. The products were quantified using proton nuclear magnetic resonance (^1^H NMR) spectroscopy (X500, Bruker Avance III HD) with water suppression. For each analysis, 0.1 mL of collected electrolyte, 0.1 mL of a DMSO as internal standard solution (0.3 mM DMSO in H_2_O) and 0.3 mL of D_2_O were mixed homogeneously. The number of moles of each liquid product (*m_l_*), including *n*‐propanol, ethanol, and acetate, was determined based on the relative integration of ^1^H NMR peaks with DMSO using the following formula:

(1) $$m_{l}=\frac{S_{l}\times H_{DMSO}}{S_{DMSO}\times H_{l}}\times c_{DMSO}\times m_{DMSO}\;$$

*S_l_* and *S_DMSO_* are the areas of the liquid product and DMSO peaks in the ^1^H NMR spectrum, respectively. *H_DMSO_* is the number of hydrogen atoms in a DMSO molecule (*H_DMSO_* = 6 for two methyl group) and *H_l_* is the number of hydrogen atoms in a molecule in liquid products (H*_n_*_‐propanol_, H_Ethanol_, and H_acetate_ = 3 for one methyl group). *c_DMSO_* is the molar concentration of the DMSO standard solution, and *m_DMSO_* is the mole of the DMSO standard solution.

The FE of products was calculated using the following equation:

(2) $$FE_{product}\left[\%\right]=\frac{e_{product}\times F\;\times \;m_{product}\;\times V_{electrolyte}}{I_{input}\times t_{reaction}}\;\times 100$$

*e_product_* is the number of electrons required to convert CO to product (*e_n‐propanol_* = 12, *e_ethanol_* = 8, *e_acetate_* = 4). F is the Faraday constant, 96,485 C mol^−1^, *m_product_* is the mole of product, *V_electrolyte_* is the volume of total electrolyte, *I_input_* is the applied current, and *t_reaction_* is reaction time.

### *Operando* Raman Measurements

Raman tests are carried out using a Renishaw inVia Raman Microscope and an in situ lens with water immersion objective (HCX APO L 63x/0.90 W UV Immersion). A custom‐made cell with a similar structure to the flow cell was used to carry out operando Raman spectroscopy. A laser wavelength of 633 nm was used as the excitation source. The laser power was kept lower than 0.20 mW in all experiments to minimize sample damage. Raman spectrometer was calibrated with the internal Si.

### Characterization

SEM images were taken using an EPIC SEM JEOL JSM‐7900FLV, and XRD was conducted with Ultima using a Cu‐Kα source. HR‐TEM bright field images were carried out on EPIC TEM JEOL ARM300F with 300 kV acceleration voltage. In order to extract lattice information of relatively thick catalyst samples, post‐processing techniques on HR‐TEM images were employed. The inherent thickness of the samples restricted conventional HR‐TEM from resolving isolated lattice information along the z‐axis, as only a single projected lattice was captured in the image. To circumvent this limitation and accurately extract grain and intragrain dimensions, inverse Fast Fourier transform (iFFT) analysis was performed based on the FFT patterns of the acquired HRTEM images. In addition, to visualize and differentiate multiple lattice domains within a single grain, color mapping analysis using the Gatan DigitalMicrograph software was conducted. XPS spectra were measured by NEXSA G2, and the binding energies were corrected for specimen charging based on 284.8 eV for the C─C bond. High‐angle annular dark‐field scanning transmission electron microscopy (HAADF‐STEM) was conducted on an aberration‐corrected JEOL ARM200CF TEM, equipped with a cold field emission gun and operated at an acceleration voltage of 200 kV. Before imaging, the sample was cleaned by vacuum annealing followed by plasma treatment using the SBT Plasma Cleaner.

## Conflict of Interest

The authors declare no conflict of interest.

## Supporting information



Supporting Information

## Data Availability

The data that support the findings of this study are available from the corresponding author upon reasonable request.
