# Supplementary material for: Copper Catalysts Inherit and Retain Precatalyst Morphology in Extended CO Electroreduction to n‐Propanol
Source: Adv Mater. 2025 Aug 23;37(45):e08900. doi: 10.1002/adma.202508900 (PMC12617019; doi:10.1002/adma.202508900)
Supplement: Supplementary file 1 — Supporting Information [file ADMA-37-e08900-s001.pdf]

ADVANCED MATERIALS

Supporting Information

for *Adv. Mater.*, DOI 10.1002/adma.202508900

Copper Catalysts Inherit and Retain Precatalyst Morphology in Extended CO Electroreduction to *n*-Propanol

Ji-Yoon Song, Jianan Erick Huang, Hyeong Woo Ban, Qiu-Cheng Chen, Yali Ji, Shuang Yang, Yong Wang, Yunsung Yoo, Hyun Seung Jung, Jiachen Li, Heejong Shin, Ke Xie and Edward H. Sargent**

Supporting Information

Copper Catalysts Inherit and Retain Precatalyst Morphology in Extended CO Electroreduction to *n*-Propanol

Ji-Yoon Song, Jianan Erick Huang, Hyeong Woo Ban, Qiu-Cheng Chen, Yali Ji, Shuang Yang, Yong Wang, Yunsung Yoo, Hyun Seung Jung, Jiachen Li, Heejong Shin, Ke Xie and Edward H. Sargent**

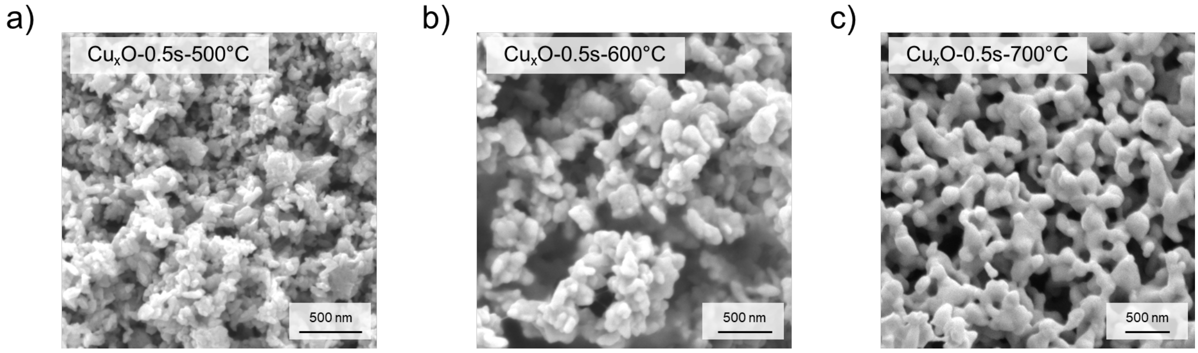

Figure S1. SEM images of a) Cu_xO-0.5s-500°C, b) Cu_xO-0.5s-600°C, c) Cu_xO-0.5s-700°C.

At 500 °C, Cu_xO-0.5s-500°C exhibited a morphology composed of discrete, non-interconnected particles. Upon increasing the temperature to 600 °C, partial aggregation of individual particles was observed. At 700 °C, the Cu oxide domains underwent significant sintering, forming a continuous network in which the particles were fully interconnected, thereby enabling the formation of a free-standing catalyst structure.

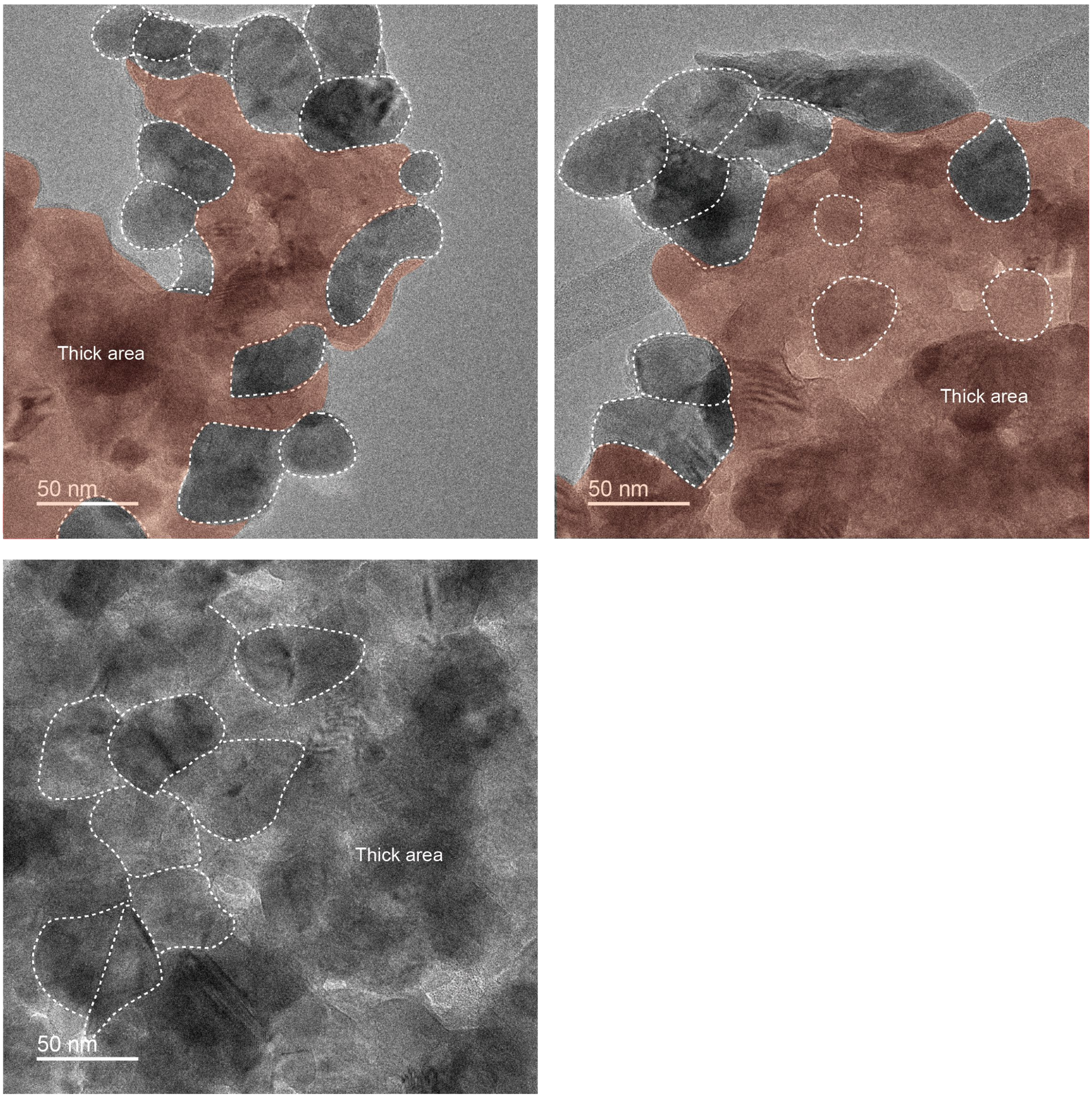

Figure S2. HR-TEM images of Cu_xO-0.5s (before electroreduction) at different spots for the grain size distribution.

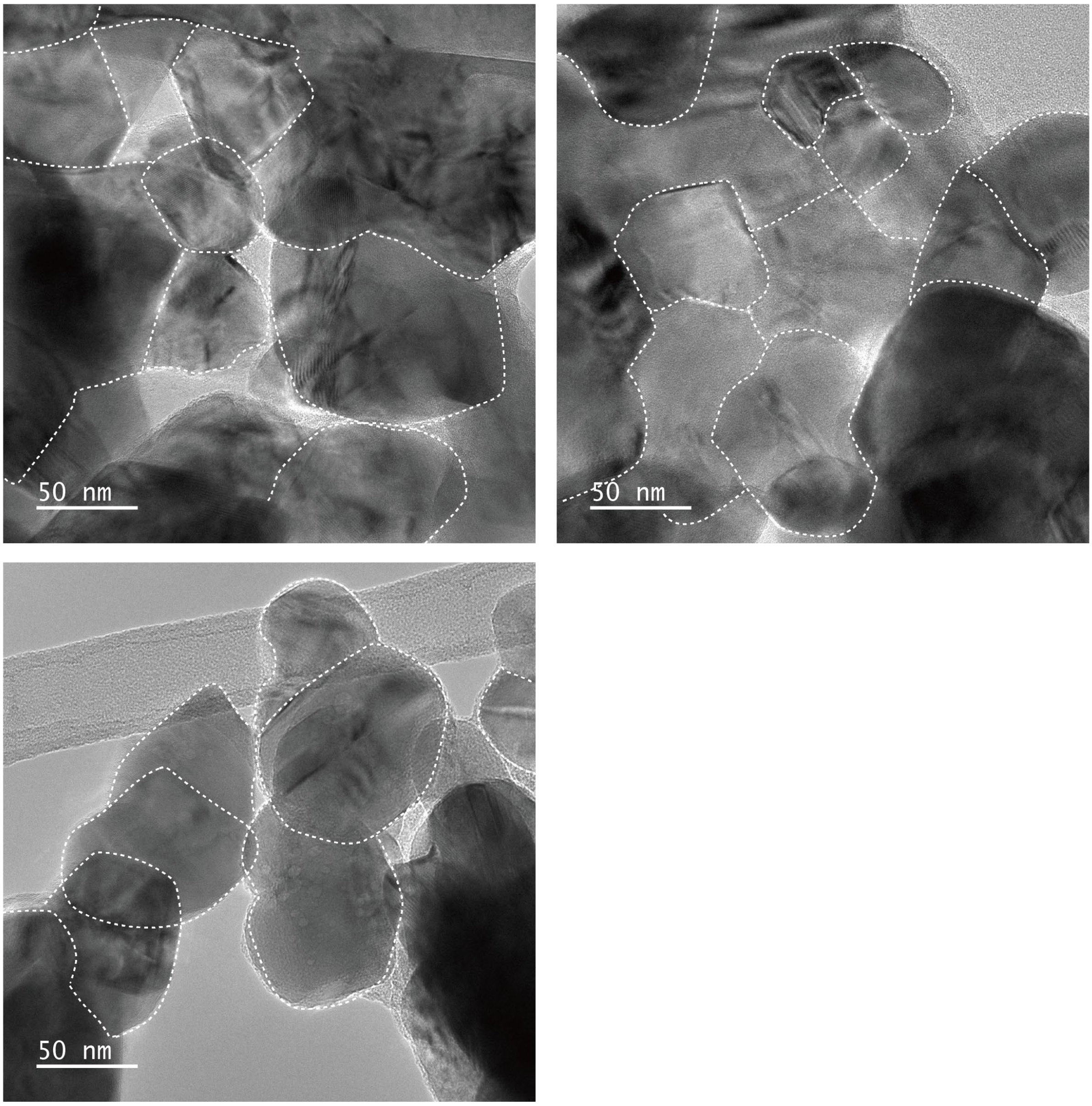

Figure S3. HR-TEM images of Cu_xO-3s (before electroreduction) at different spots for the grain size distribution.

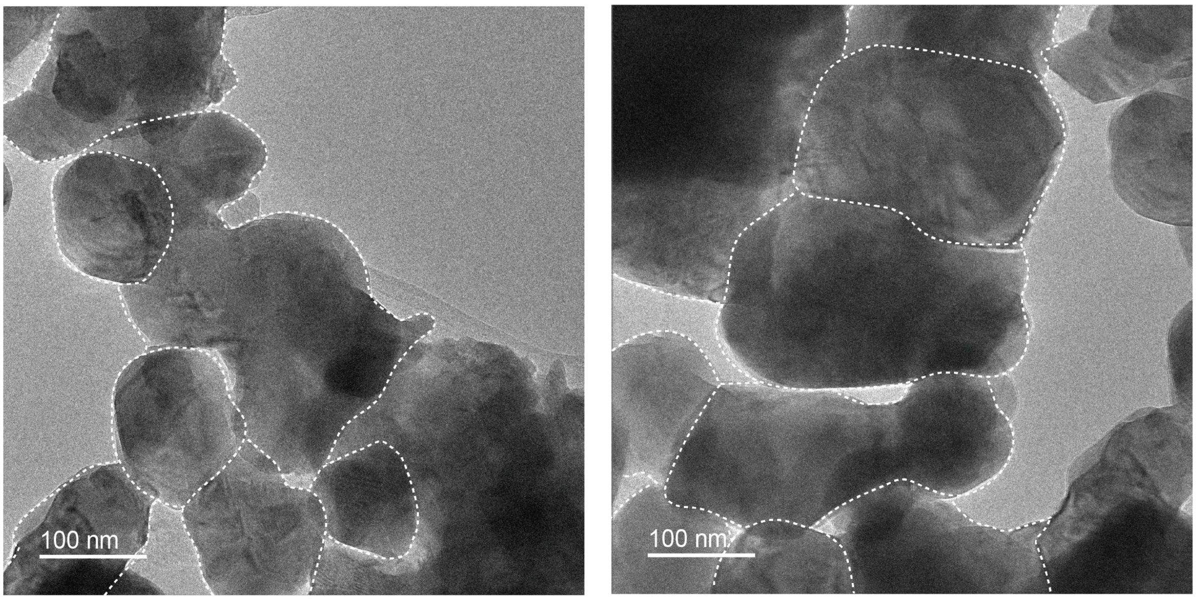

Figure S4. HR-TEM images of Cu_xO-10s (before electroreduction) at different spots for the grain size distribution.

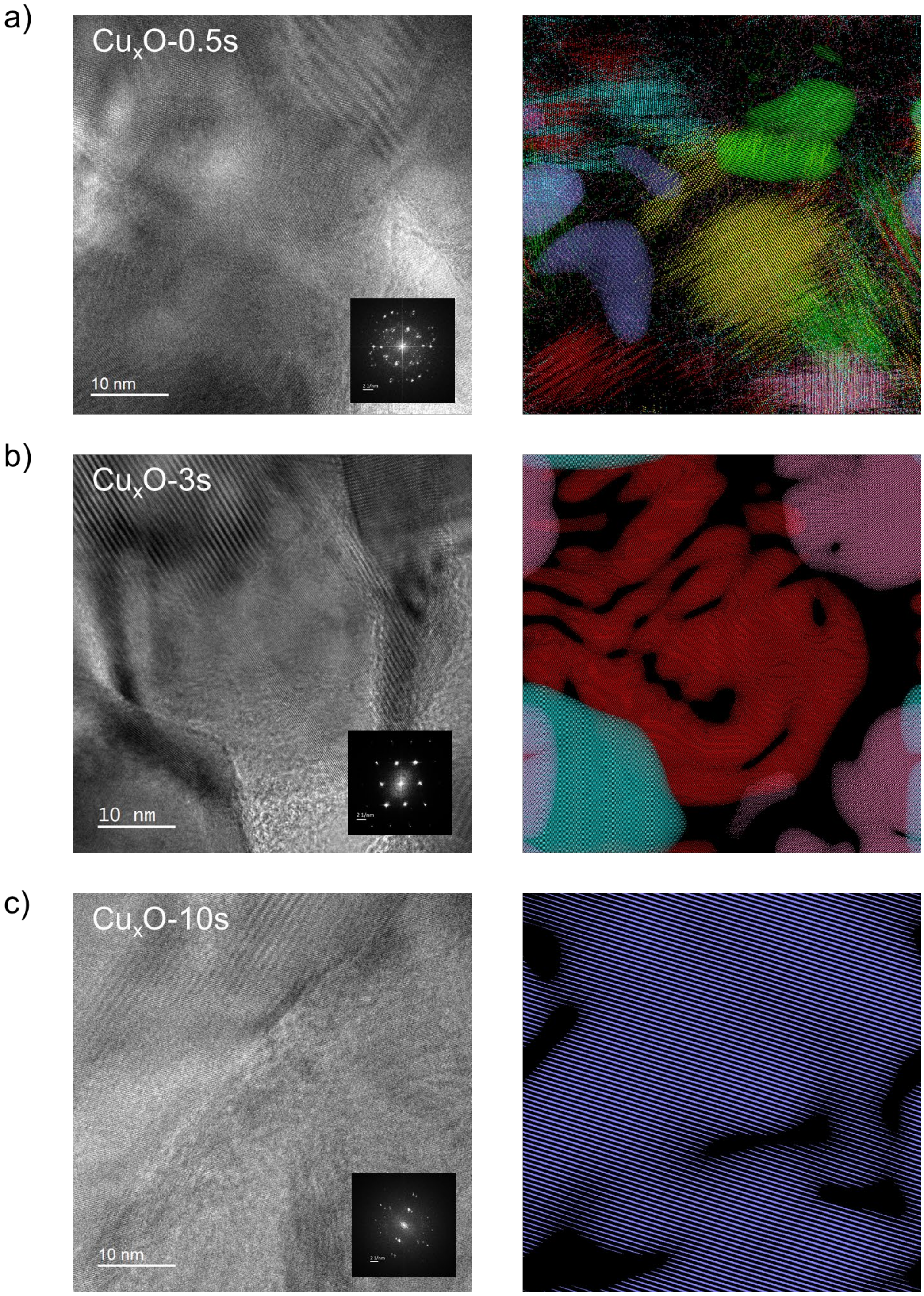

Figure S5. HR-TEM images and corresponding Cu_xO grains (inset: FFT patterns)

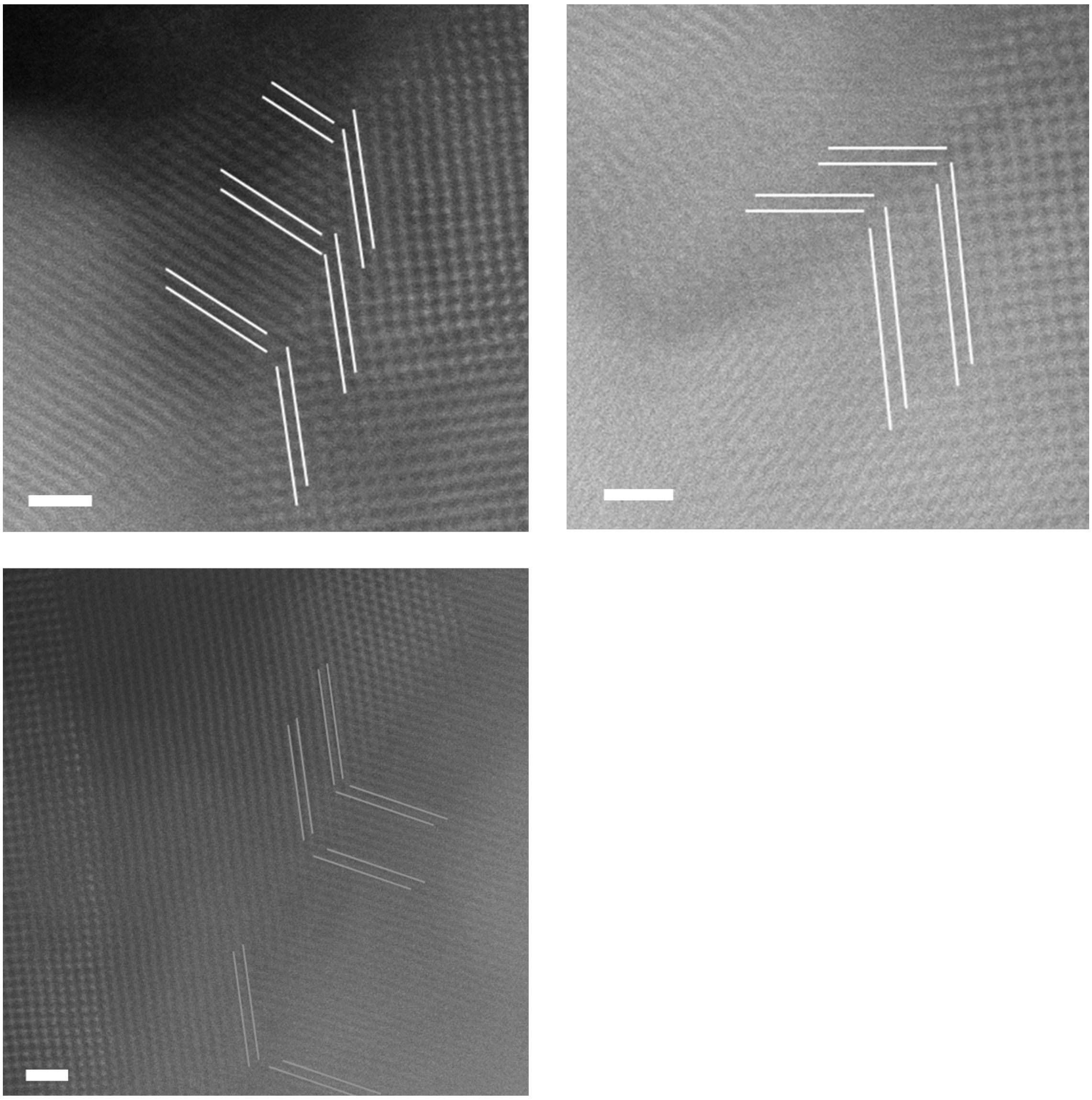

Figure S6. STEM images of CuOx-0.5s (scale bars: 1 nm).

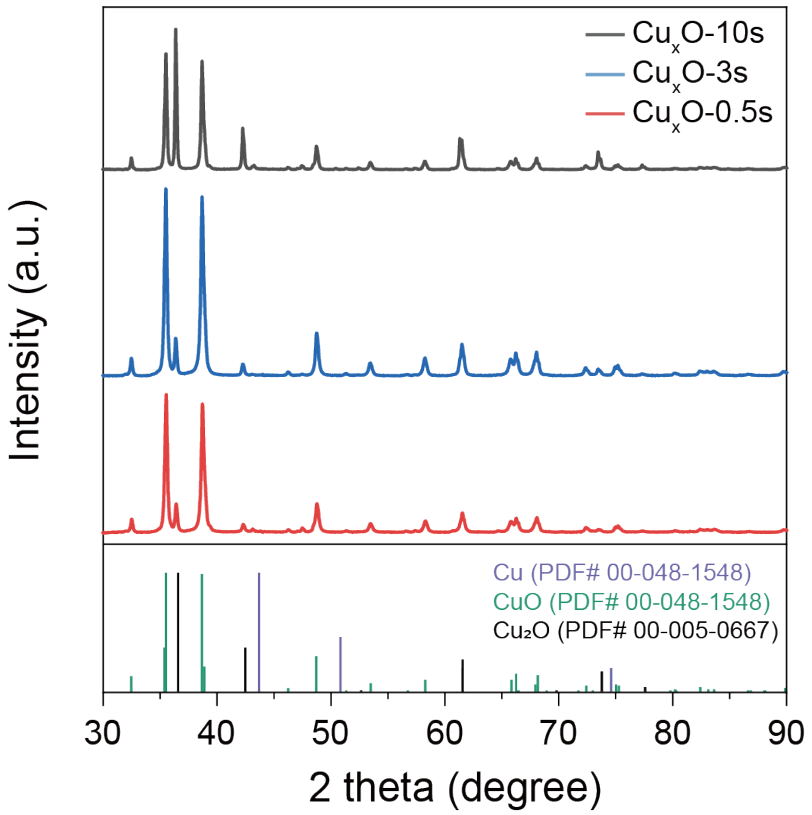

Figure S7. XRD patterns of precatalysts (Cu_xO-*x*s) prior to electroreduction.

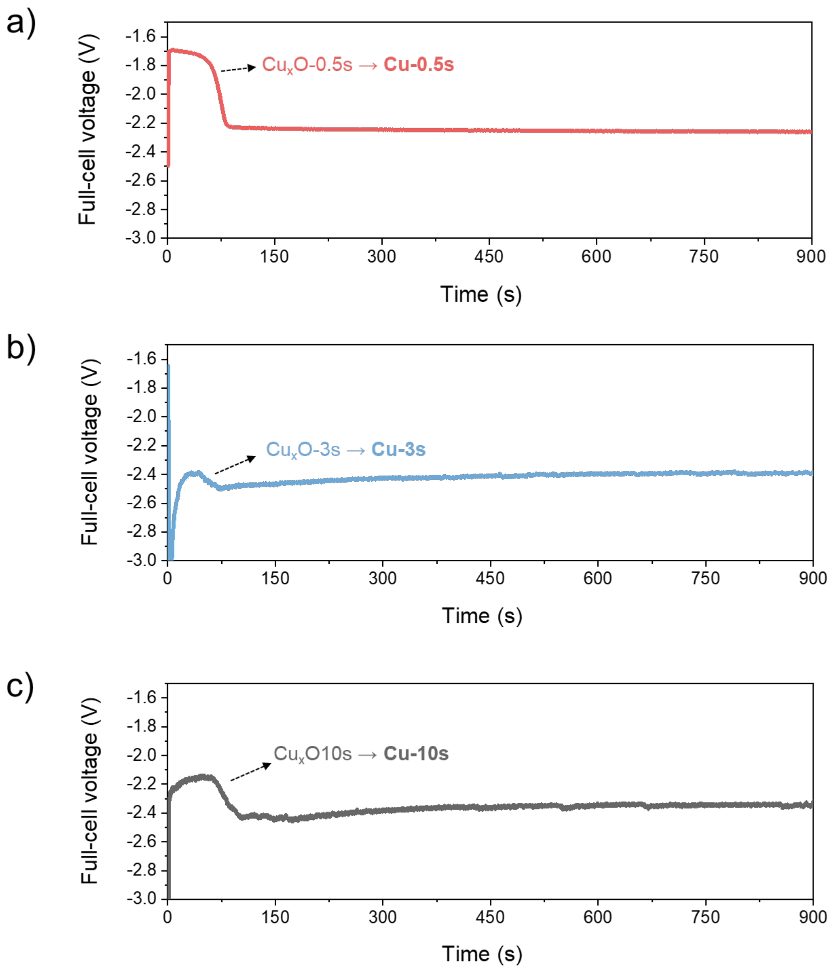

Figure S8. Chronopotentiometry (CP) profiles of a) Cu-0.5s, b) Cu-3s, and c) Cu-10s electrodes during electroreduction from Cu_xO to metallic Cu under CO feeding conditions in a MEA electrolyser.

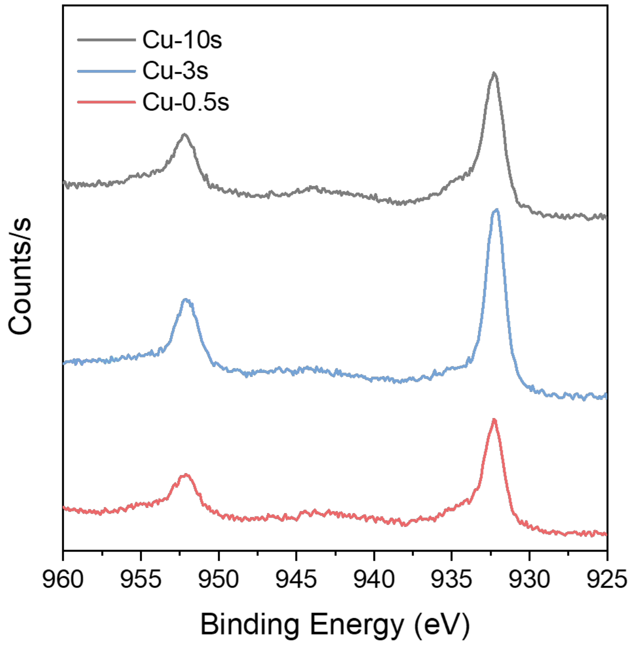

Figure S9. XPS Cu *2p* high-resolution spectra of Cu-0.5, 3, and 10s.

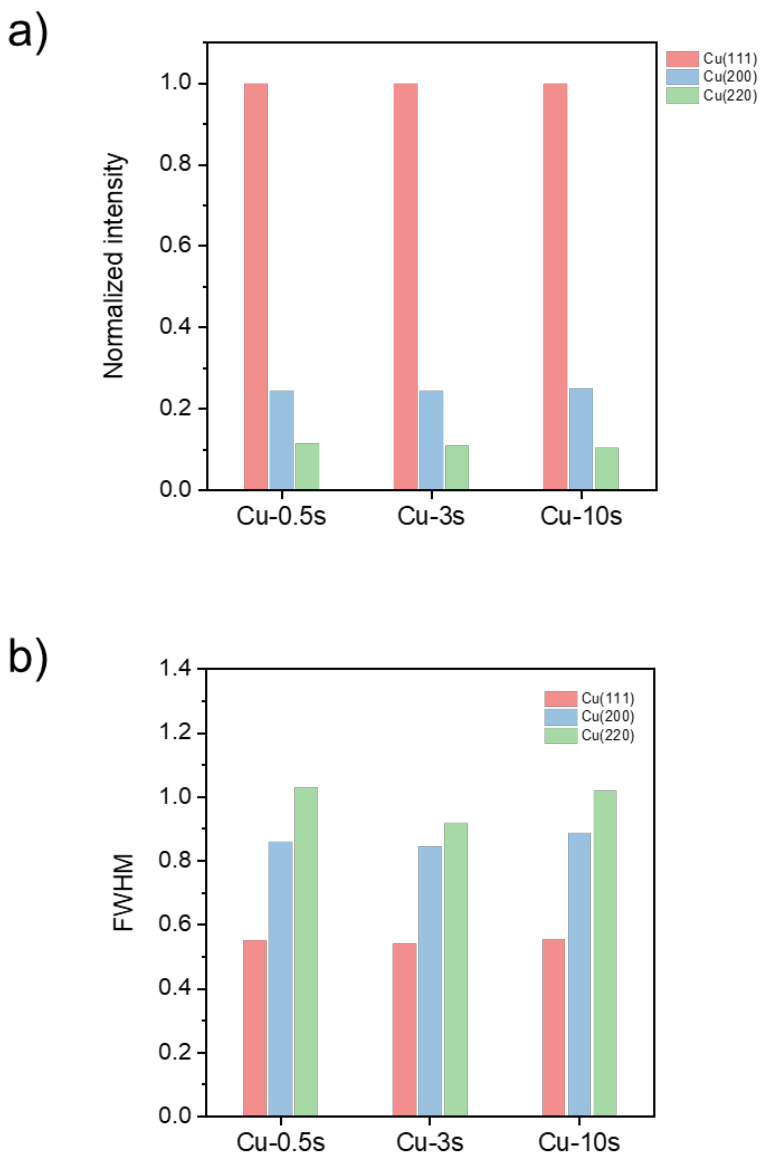

Figure S10. a) The facet ratio and b) the full width at half maximum (HWHM) of metallic Cu of Cu-0.5, 3, and 10s (after electroreduction) from XRD patterns in **Figure 2a**.

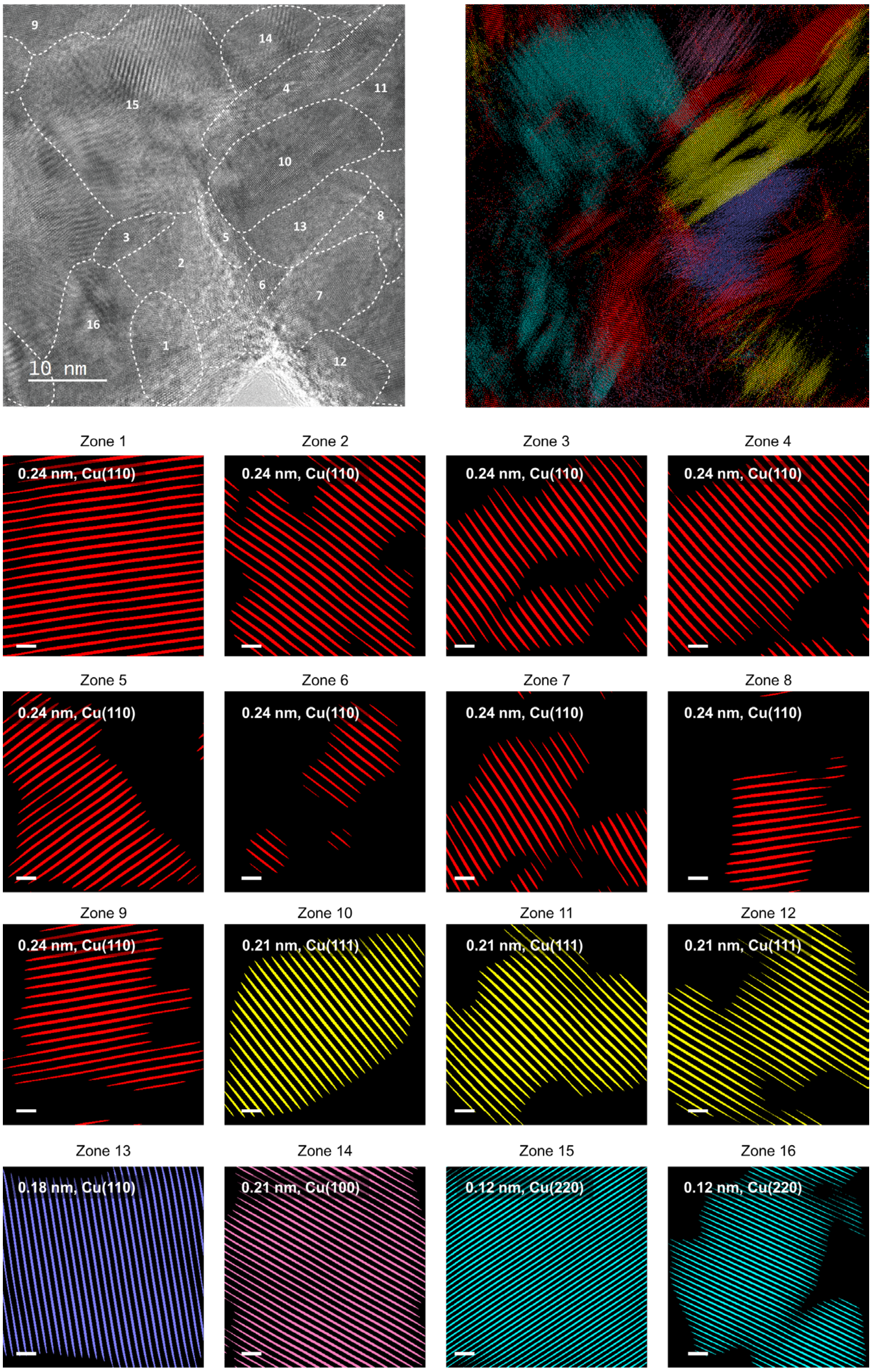

Figure S11. Intragrain structure of Cu-0.5s in **Figure 2b** and corresponding lattice spacings extracted from TEM analysis.

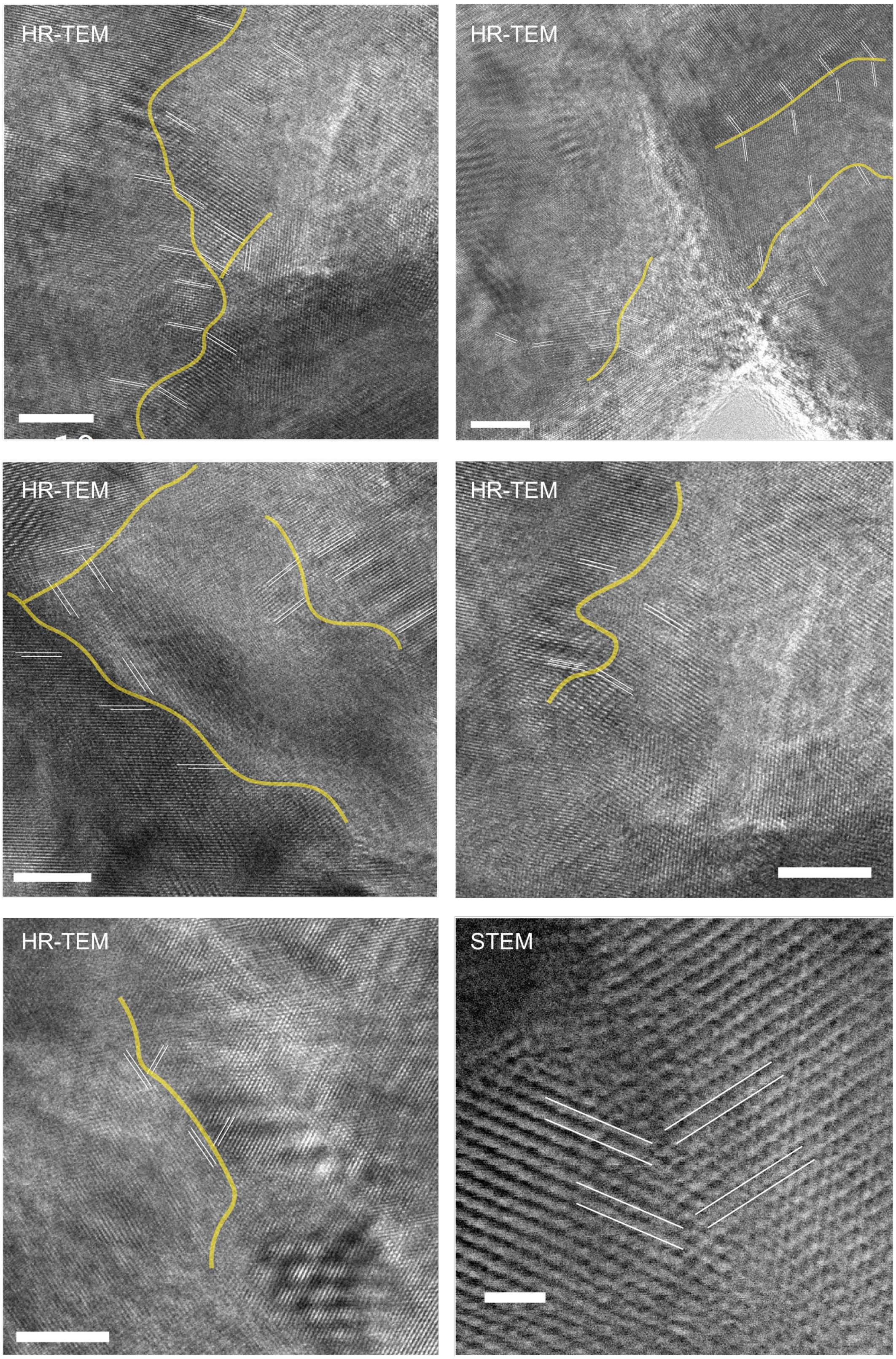

Figure S12. HR-TEM and STEM images of Cu-0.5s (scale bars: 5 nm for HR-TEM, and 1 nm for STEM). Atomic-scale intragrain boundaries are indicated by yellow dashed lines.

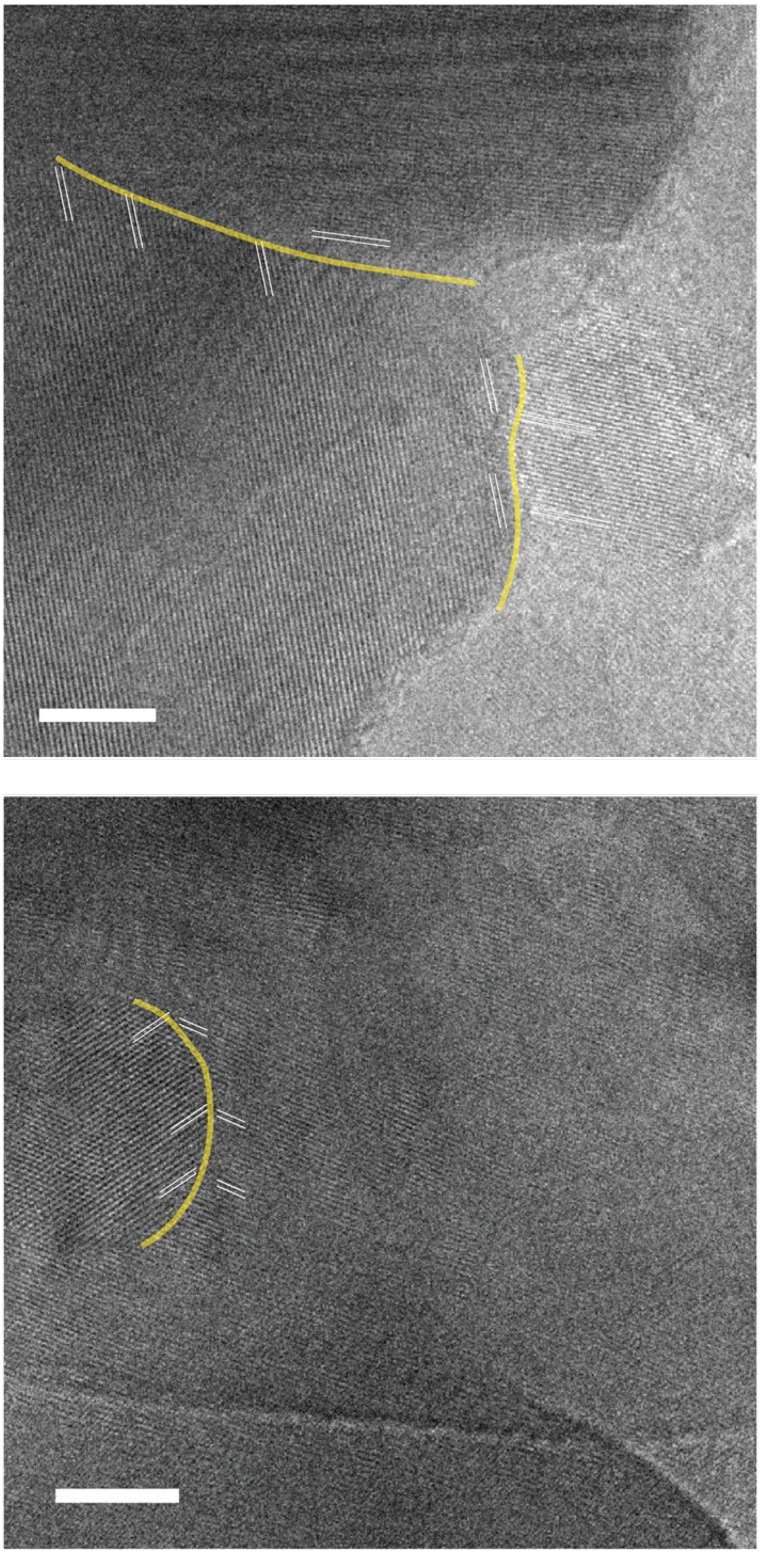

Figure S13. HR-TEM images of Cu-3s (scale bars: 5 nm). Atomic-scale intragrain boundaries are indicated by yellow dashed lines.

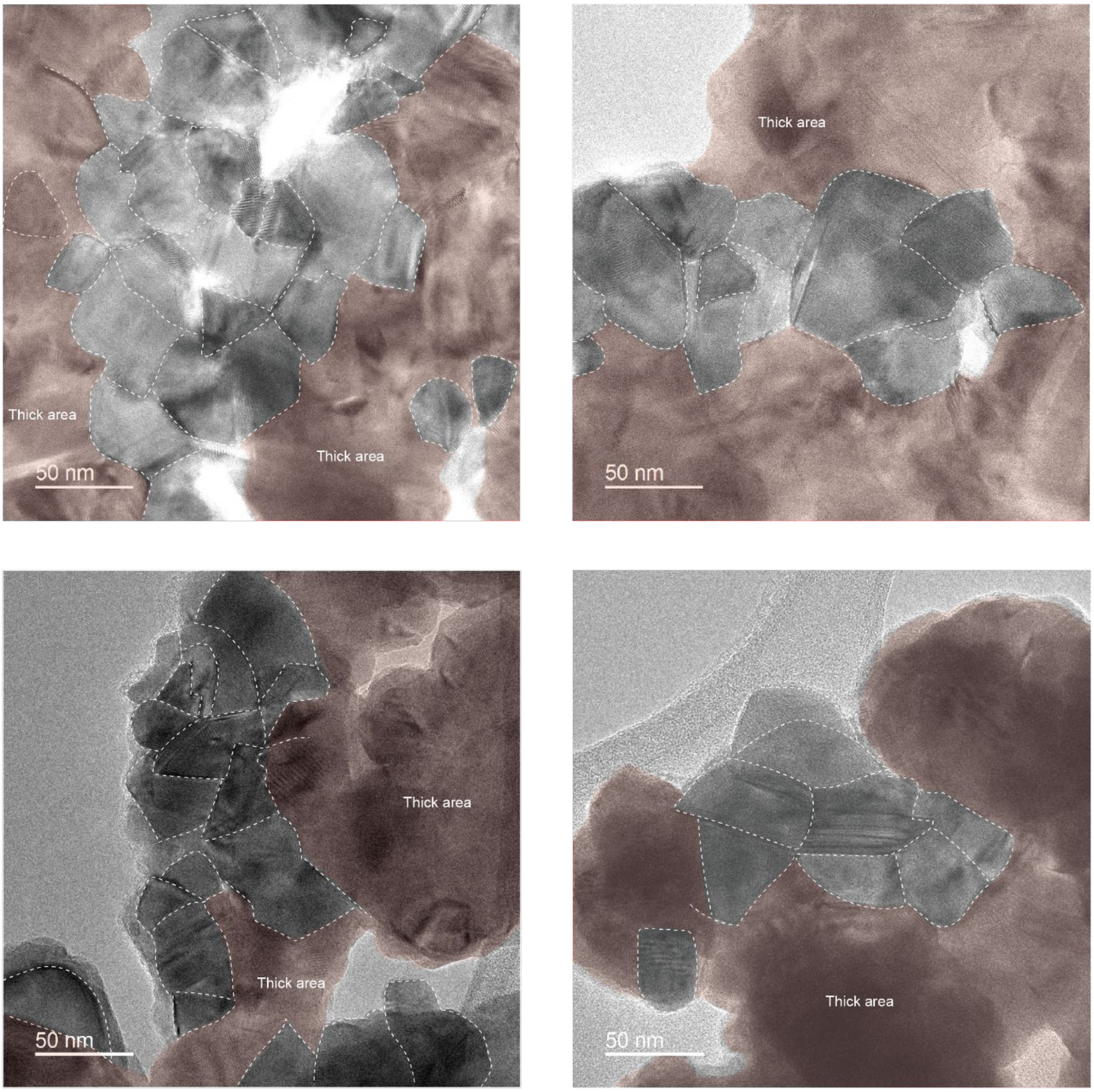

Figure S14. HR-TEM images of Cu-0.5s (after electroreduction) at different spots for the grain size distribution.

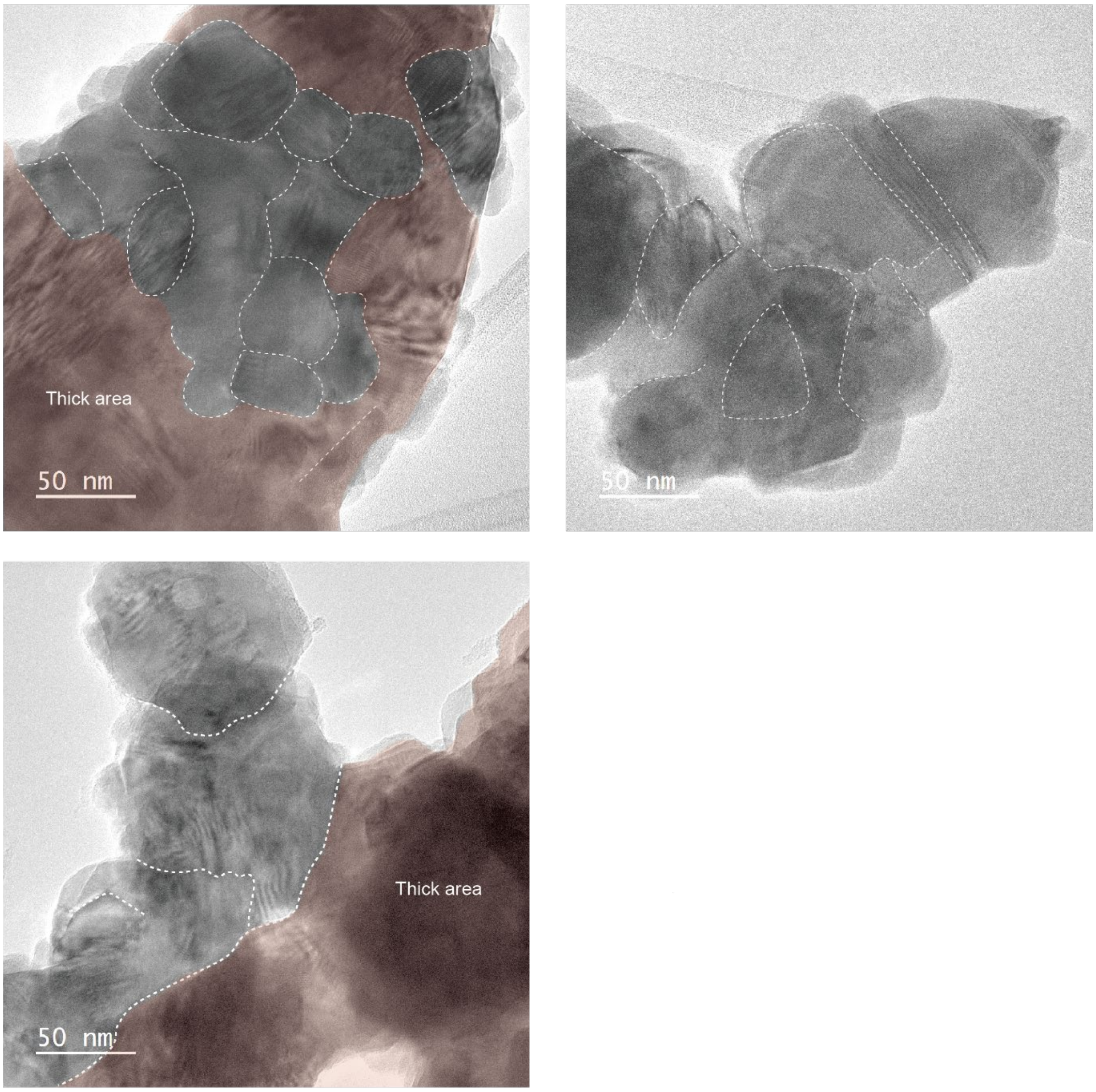

Figure S15. HR-TEM images of Cu-3s (after electroreduction) at different spots for the grain size distribution.

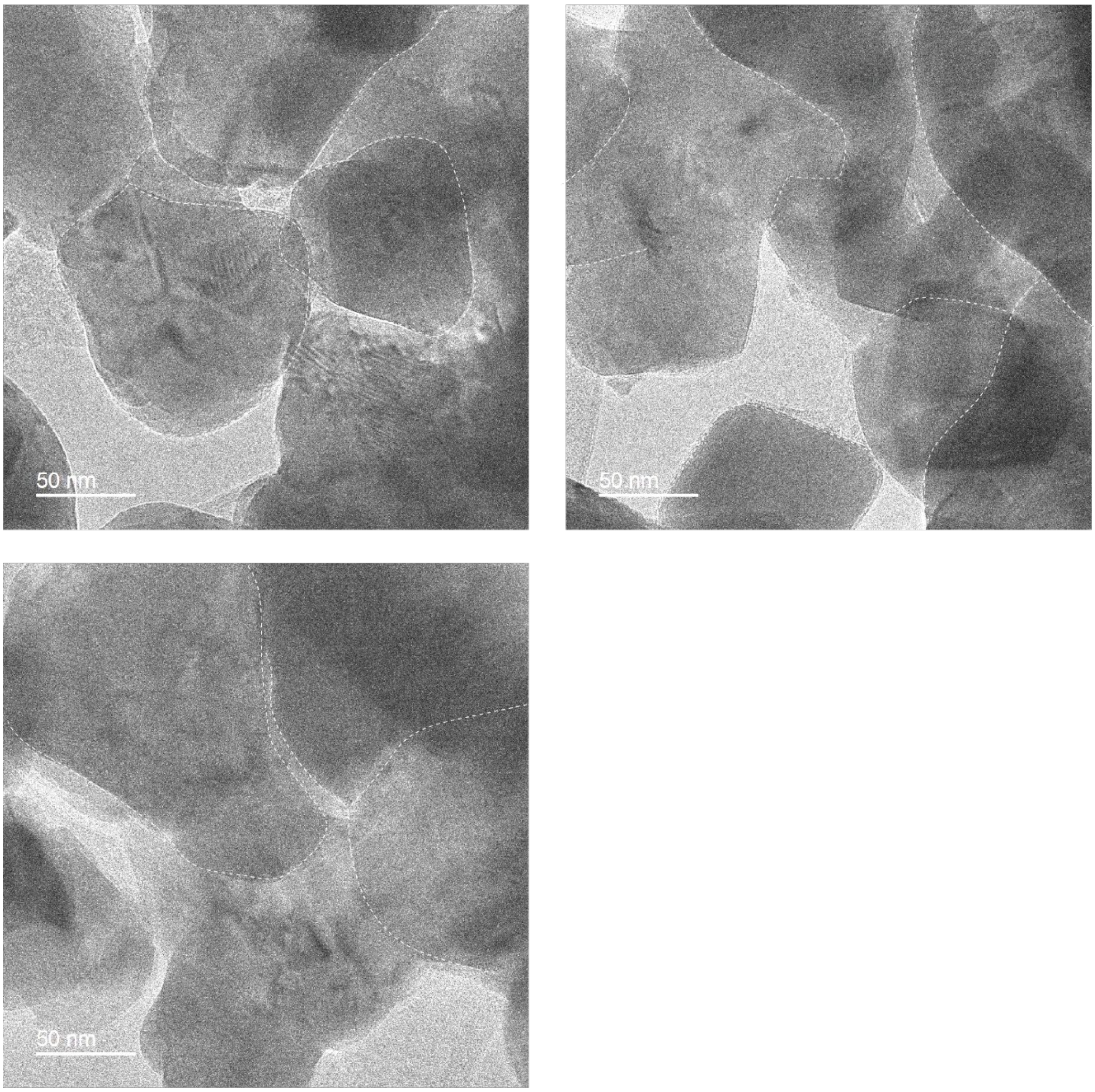

Figure S16. HR-TEM images of Cu-10s (after electroreduction) at different spots for the grain size distribution.

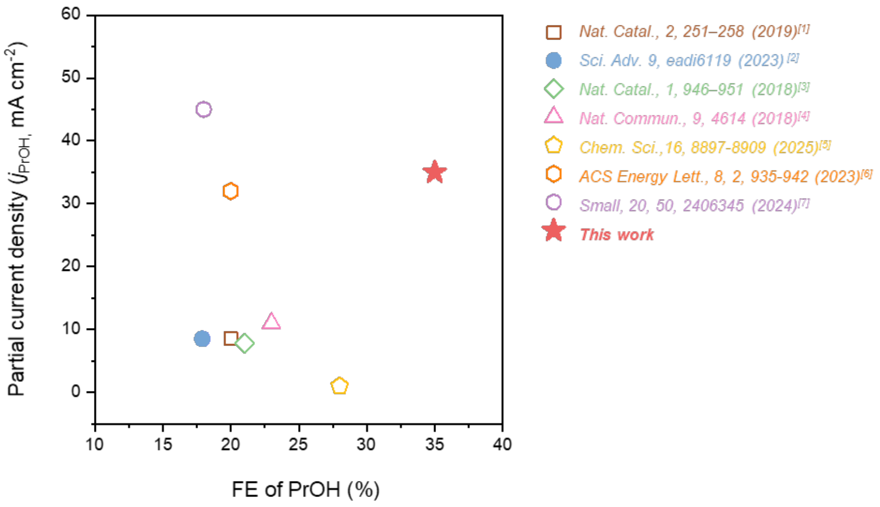

Figure S17. States-of-arts of CO-to-propanol of monometallic Cu catalysts.

Filled markers represent studies in which stability tests were conducted, while hollow markers correspond to reports where only performance tests were performed without long-term stability evaluation.

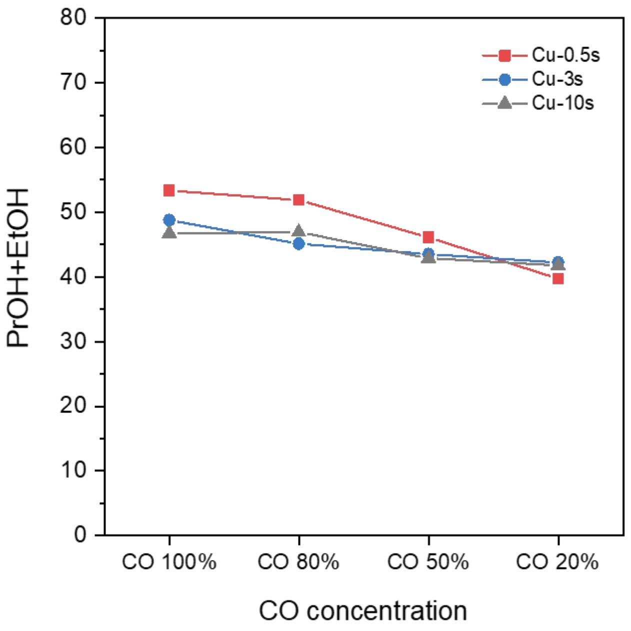

Figure S18. Combined FEs of alcohols (ethanol and *n*-propanol) depending on CO concentration of Cu-0.5, 3 and 10s.

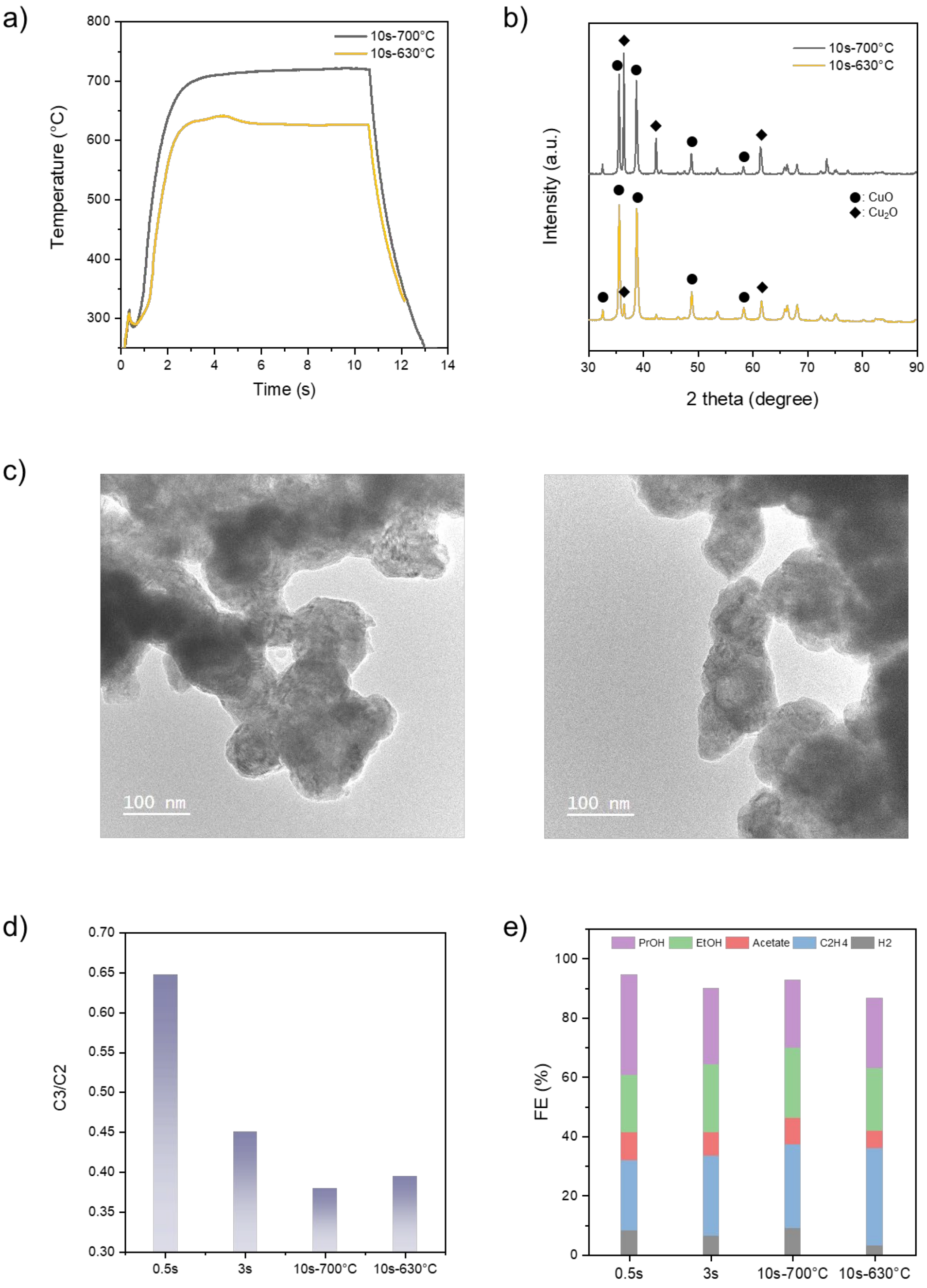

Figure S19. Initial oxidation state dependence of Cu_xO-10s. a) temperature profile, b) XRD patterns, c) TEM images of Cu-10s-630°C, d) C3/C2 product ratio and e) Faradaic efficiencies after CORR.

To investigate the effect of Joule-heating time, we synthesized CuxO-10s sample, which is the longest thermal exposure and exhibited a Cu2O-dominant phase, as shown in Figure S6. To decouple the influence of initial oxidation state from structural parameters such as grain size, we synthesized a control sample, Cu_xO-10s-630°C, by applying the lower current to reach 630°C for 10 s (**Figure S20a**). Cu_xO-10s-630°C sample showed a similar oxidation state to CuO-dominant samples like Cu_xO-0.5s and 3s while maintaining a comparable grain structure like CuxO-10s-700°C (**Figure S20b and c**). TEM images confirmed that this sample retained a similar average grain size (105.6 ± 18.0 nm) and interconnected morphology (**Figure S20c**).

Electrochemical CORR was conducted under same conditions revealed that the Cu-10s-630°C sample exhibited C3/C2 ratio and FE of propanol comparable to those of the Cu-10s-700°C sample. Importantly, the trend in C3/C2 ratio with respect to grain/intragrain size kept consistent (**Figure S20d and e**). While minor effects from initial oxidation state variations cannot be entirely ruled out, our comparative experiments indicate that the observed performance trends are predominantly governed by grain structure.

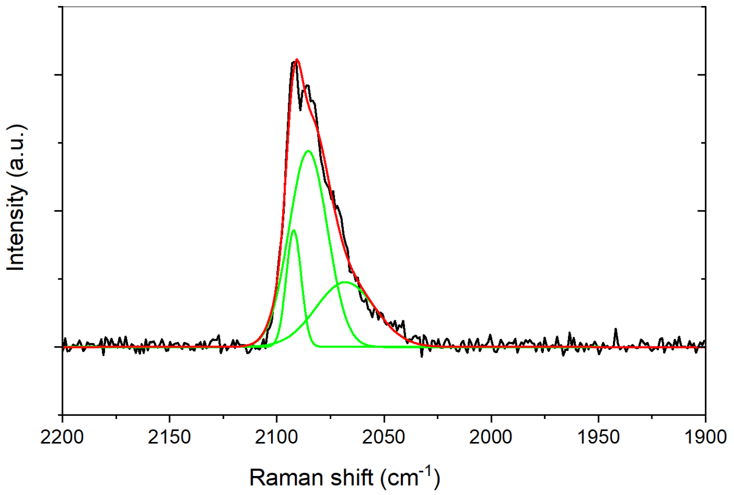

Figure S20. Deconvolution of in situ Raman spectra in the range of 1900—2100 cm^{-1} of Cu-0.5s.

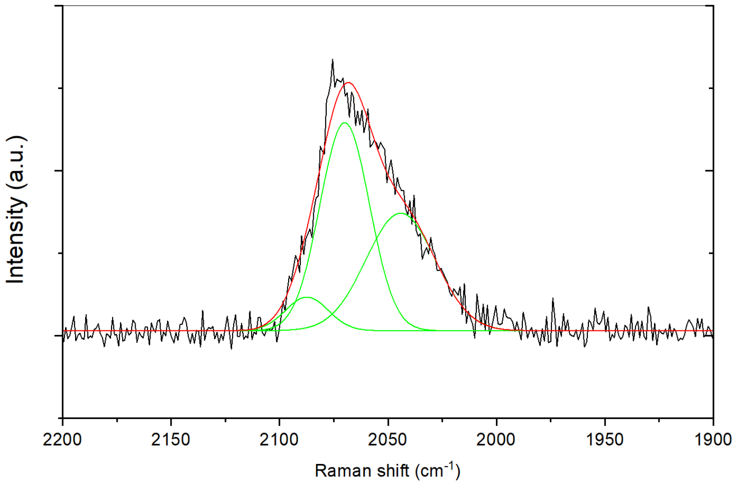

Figure S21. Deconvolution of in situ Raman spectra in the range of 1900—2100 cm^{-1} of Cu-3s.

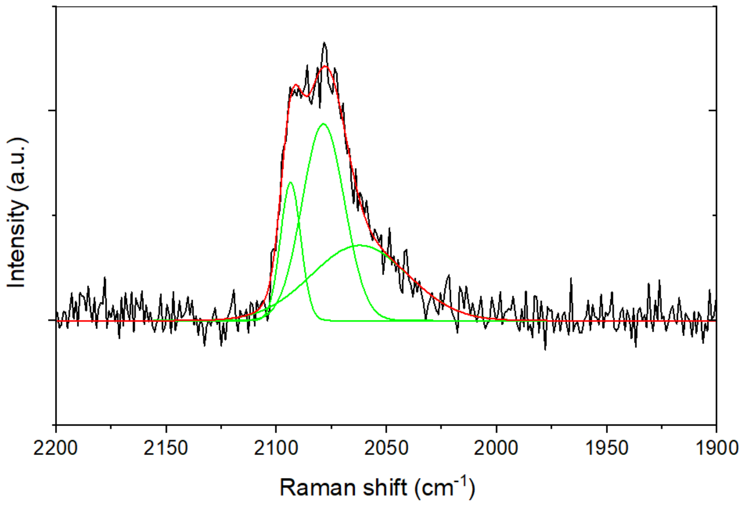

Figure S22. Deconvolution of in situ Raman spectra in the range of 1900—2100 cm^{-1} of Cu-10s.

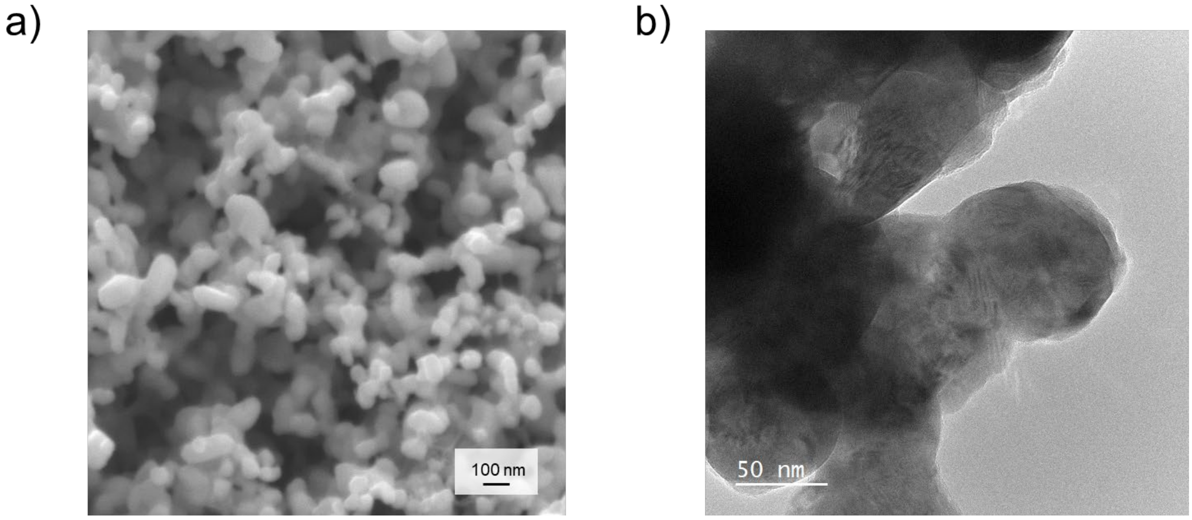

Figure S23. a) SEM image and b) TEM image of Cu-0.5s-60K/s.

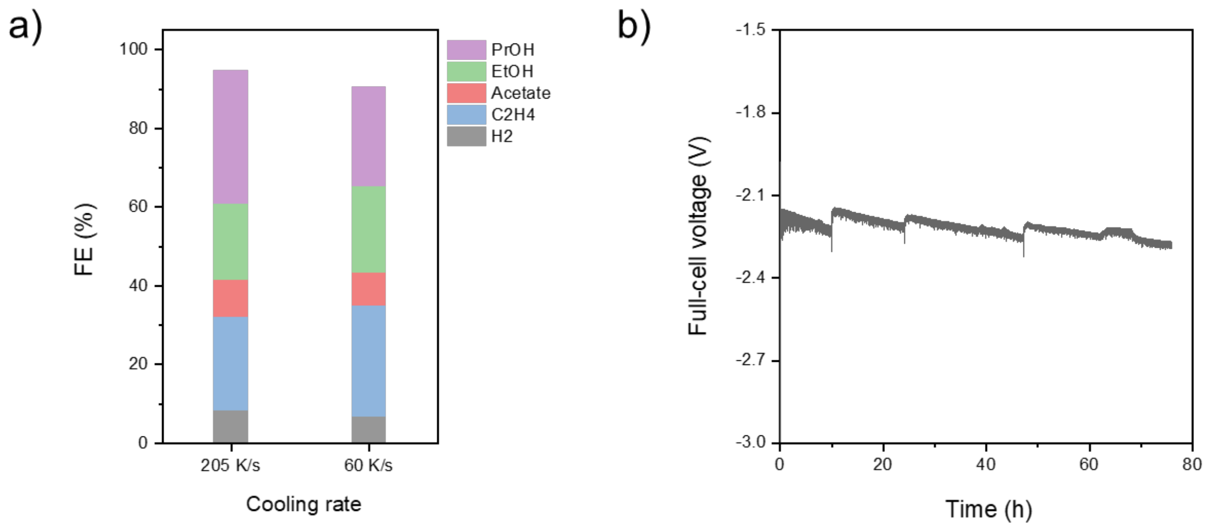

Figure S24. CORR operation results of Cu-0.5s-60K/s. a) Faradaic efficiencies of Cu-0.5s (205 K/s) and Cu-0.5s-60K/s and b) chronopotentiometry (CP) profile during long-term stability test at -100 mA cm^{-2}

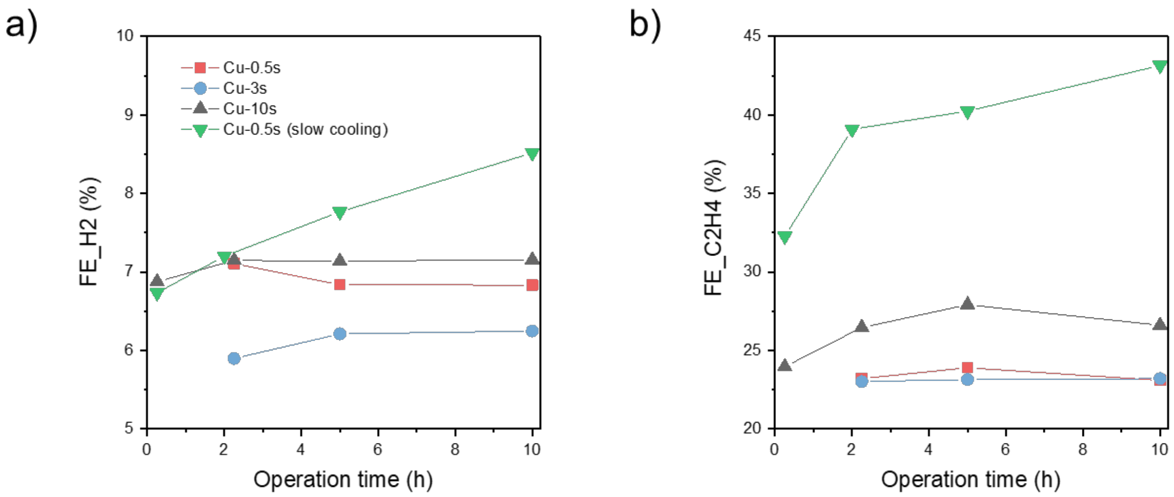

Figure S25. Faradaic efficiencies of a) H_2 and b) C_2H_4 as a function of CORR operation time.

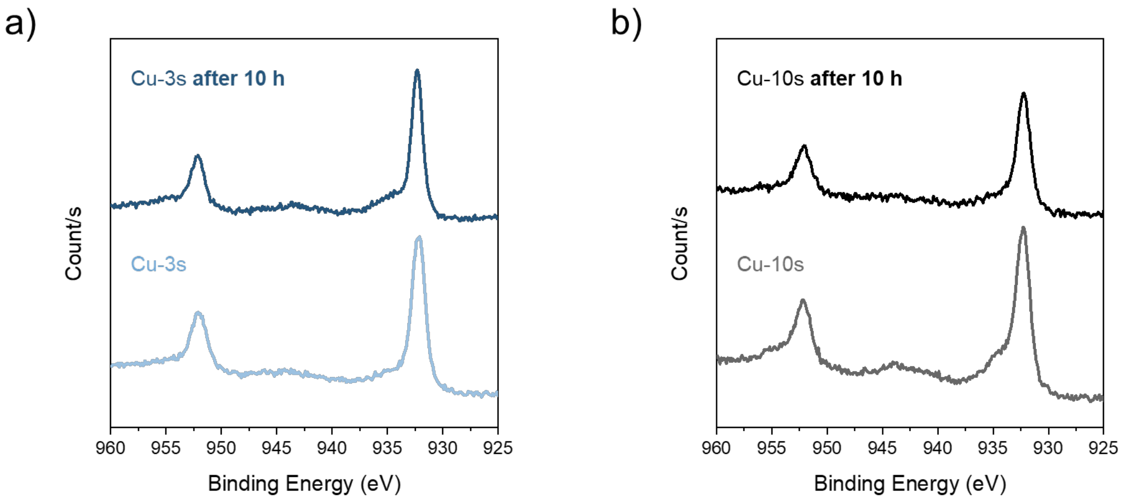

Figure S26. Cu *2p* XPS high-resolution spectra after 10 hours stability test of a) Cu-3s and b) Cu-10s.

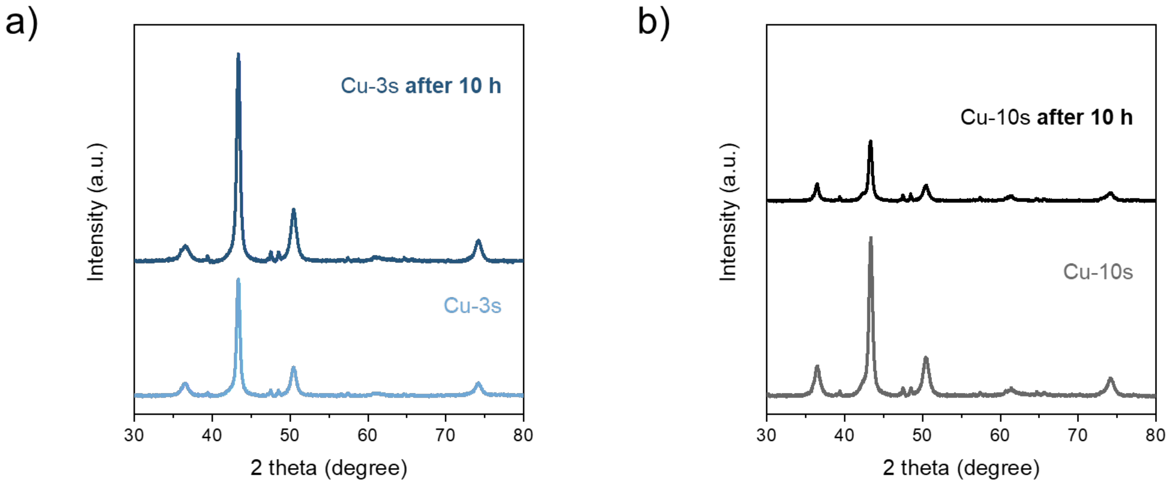

Figure S27. XRD patterns after 10 hours stability test of a) Cu-3s and b) Cu-10s.

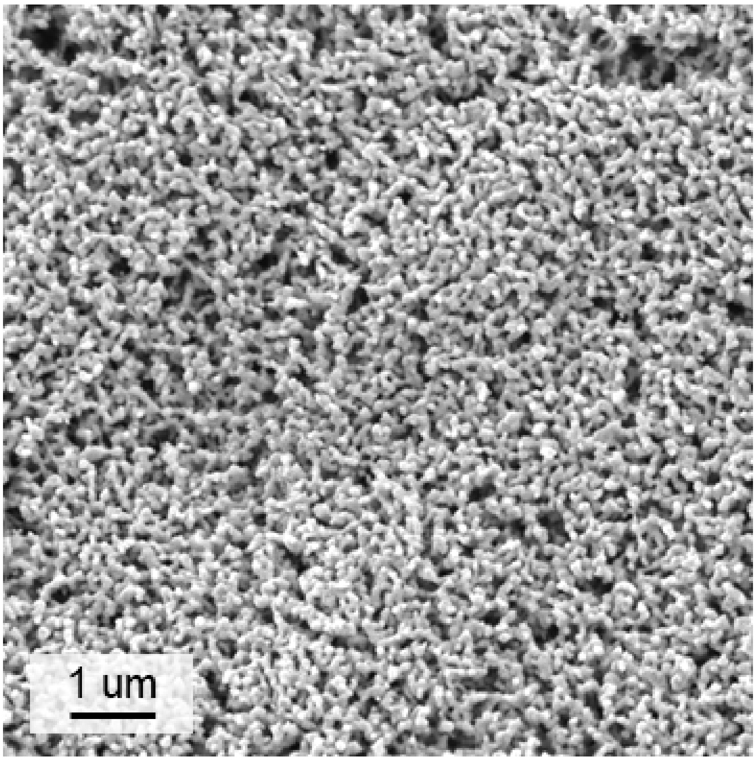

Figure S28. SEM image after 10 h stability test of Cu-3s.

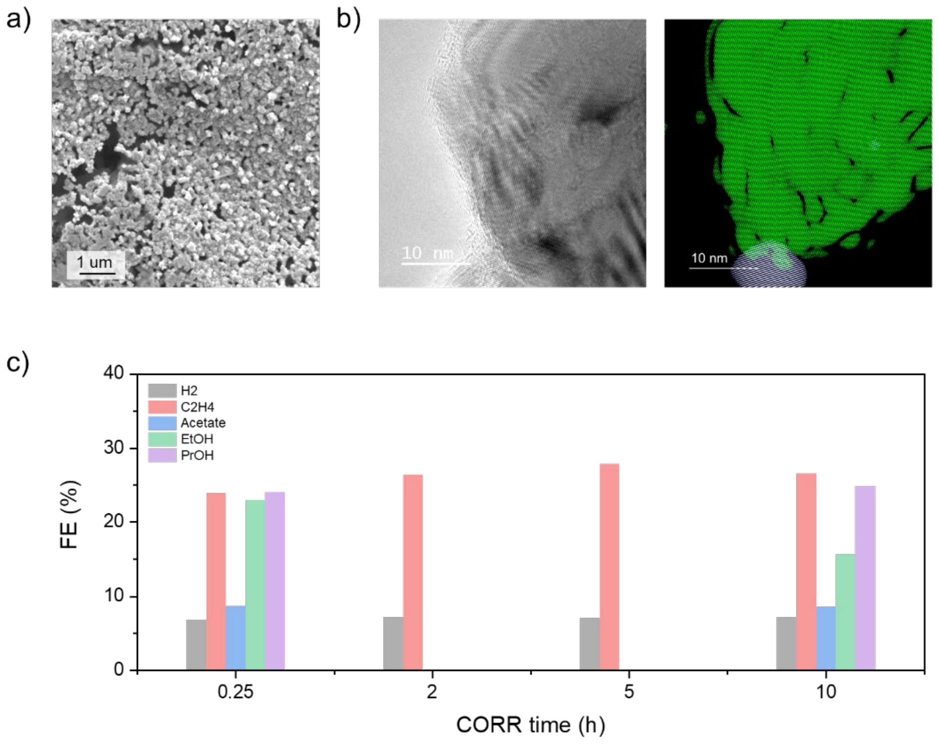

Figure S29. a) SEM image, b) TEM images after 10 h stability test and c) CORR performance during 10 h of Cu-10s.

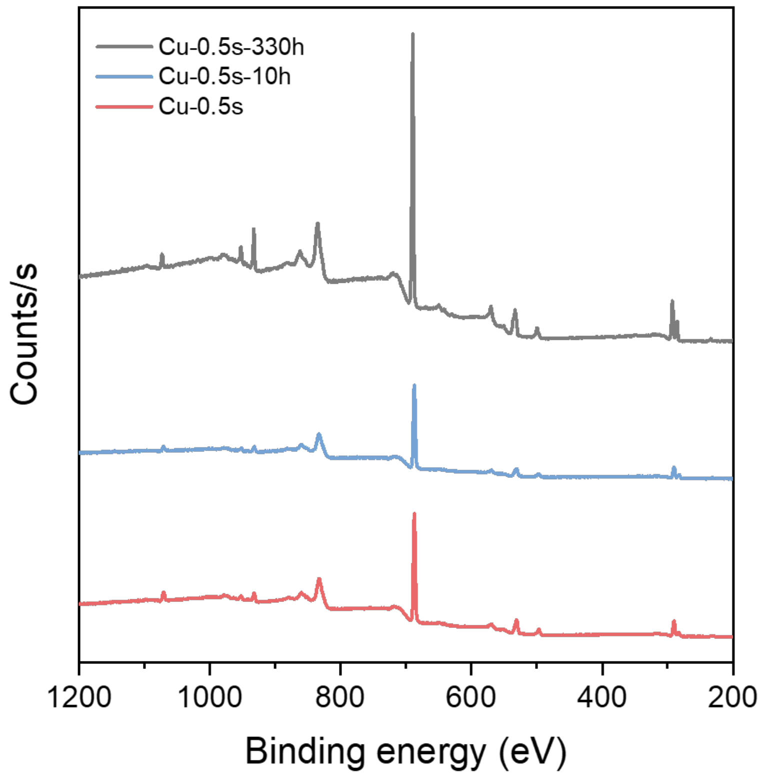

Figure S30. XPS survey scan spectra of Cu-0.5s after stability test.

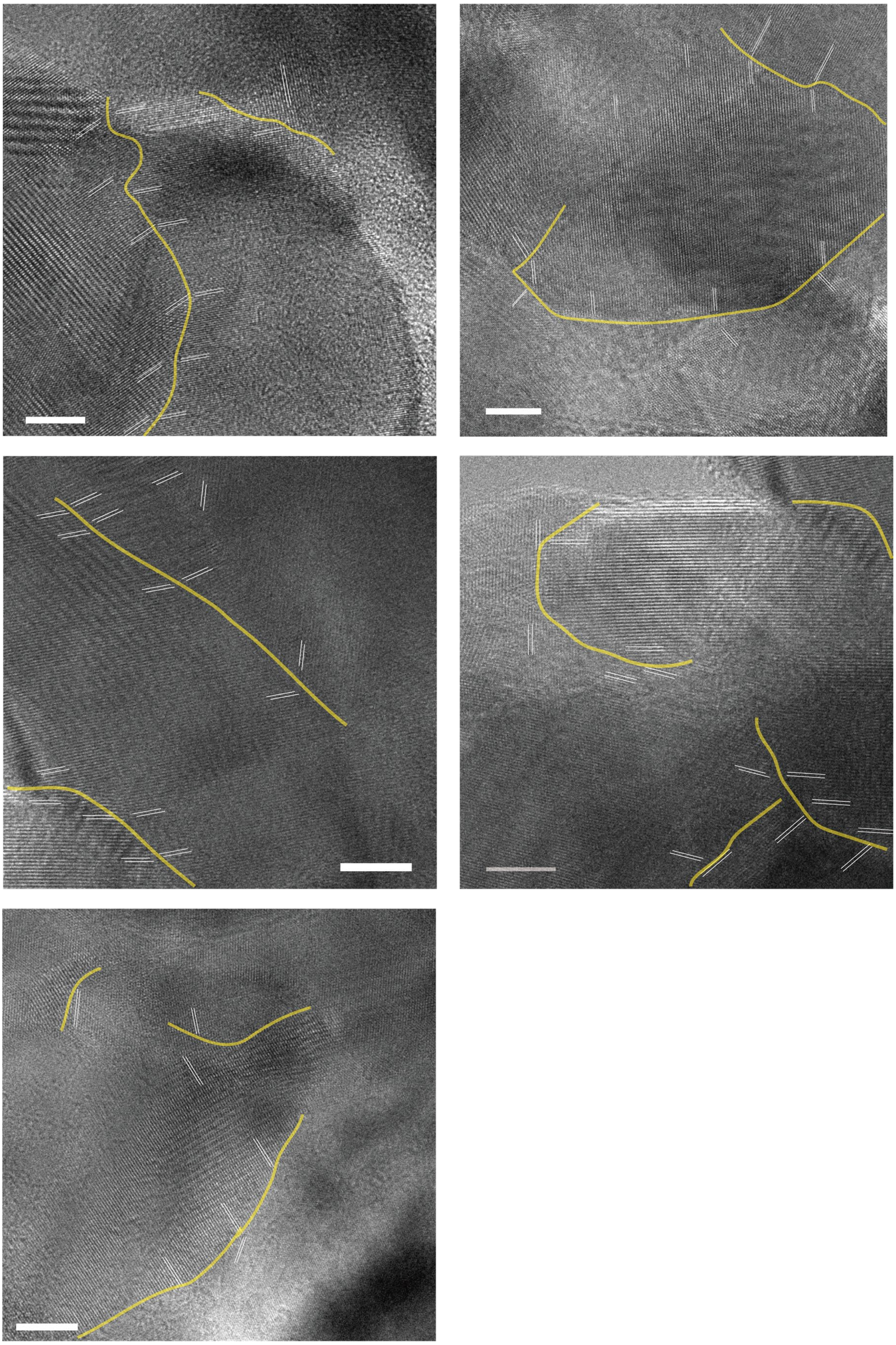

Figure S31. HR-TEM images of Cu-0.5s after 330 h operation (scale bars: 5 nm). Atomic-scale intragrain boundaries are indicated by yellow dashed lines.

References

[1] Y. J. Pang, J. Li, Z. Y. Wang, C. S. Tang, P. L. Hsieh, T. T. Zhuang, Z. Q. Liang, C. Q. Zou, X. Wang, P. De Luna, J. P. Edwards, Y. Xu, F. W. Li, C. T. Dinh, M. Zhong, Y. H. Lou, D. Wu, L. J. Chen, E. H. Sargent, D. Sinton, Nat. Catal. **2019**, 2, 251.

[2] C. Long, X. L. Liu, K. W. Wan, Y. H. Jiang, P. F. An, C. Y. Yang, G. L. Wu, W. Y. Wang, J. Guo, L. Li, K. L. Pang, Q. Li, C. H. Cui, S. Q. Liu, T. Tan, Z. Y. Tang, Sci. Adv. **2023**, 9, eadi6119.

[3] T. T. Zhuang, Y. J. Pang, Z. Q. Liang, Z. Y. Wang, Y. Li, C. S. Tan, J. Li, C. T. Dinh, P. De Luna, P. L. Hsieh, T. Burdyny, H. H. Li, M. X. Liu, Y. H. Wang, F. W. Li, A. Proppe, A. Johnston, D. H. Nam, Z. Y. Wu, Y. R. Zheng, A. H. Ip, H. R. Tan, L. J. Chen, S. H. Yu, S. O. Kelley, D. Sinton, E. H. Sargent, Nat. Catal. **2018**, 1, 946.

[4] J. Li, F. L. Che, Y. J. Pang, C. Q. Zou, J. Y. Howe, T. Burdyny, J. P. Edwards, Y. H. Wang, F. W. Li, Z. Y. Wang, P. De Luna, C. T. Dinh, T. T. Zhuang, M. I. Saidaminov, S. Cheng, T. Wu, Y. Z. Finfrock, L. Ma, S. H. Hsieh, Y. S. Liu, G. A. Botton, W. F. Pong, X. Du, J. H. Guo, T. K. Sham, E. H. Sargent, D. Sinton, Nat. Commun. **2018**, 9, 4614.

[5] R. X. Qiu, L. X. Cui, L. Peng, O. A. Syzgantseva, J. R. Li, N. Fang, M. A. Syzgantseva, Y. Jiang, J. Zhang, B. X. Zhang, L. Z. Ding, Y. Y. Dong, T. W. Xue, C. Li, J. C. Dong, J. Y. Ye, I. Akpinar, S. L. Yang, J. Li, J. L. Zhang, J. F. Li, B. X. Han, Chem. Sci. **2025**, 16, 8897.

[6] S. Y. Guo, Y. C. Liu, Y. Huang, H. S. Wang, E. Murphy, L. Delafontaine, J. L. Chen, I. V. Zenyuk, P. Atanassov, ACS Energy Lett. **2023**, 8, 935.

[7] Y. Q. Yan, K. H. Liu, C. Yang, Y. S. Chen, X. M. Lv, C. J. Hu, L. J. Zhang, G. F. Zheng, Small **2024**, 20, 2406345.
